# Functional characterization of the gonococcal polyphosphate pseudo-capsule

**DOI:** 10.1371/journal.ppat.1011400

**Published:** 2023-05-22

**Authors:** Benedetta Manca, Giada Buffi, Greta Magri, Mariangela Del Vecchio, Anna Rita Taddei, Alfredo Pezzicoli, Maria Giuliani

**Affiliations:** 1 Pharmacy and Biotechnology Department (FaBiT), University of Bologna, Bologna, Italy C/O GSK, Siena, Italy; 2 GSK, Siena, Italy; 3 Centre for High Instruments, Electron Microscopy Section, University of Tuscia, Viterbo, Italy; University of Oxford, UNITED KINGDOM

## Abstract

*Neisseria gonorrhoeae* is an exclusively human pathogen able to evade the host immune system through multiple mechanisms. Gonococci accumulate a large portion of phosphate moieties as polyphosphate (polyP) on the exterior of the cell. Although its polyanionic nature has suggested that it may form a protective shield on the cell surface, its role remains controversial. Taking advantage of a recombinant His-tagged polyP-binding protein, the presence of a polyP pseudo-capsule in gonococcus was demonstrated. Interestingly, the polyP pseudo-capsule was found to be present in specific strains only. To investigate its putative role in host immune evasion mechanisms, such as resistance to serum bactericidal activity, antimicrobial peptides and phagocytosis, the enzymes involved in polyP metabolism were genetically deleted, generating mutants with altered polyP external content. The mutants with lower polyP content on their surface compared to the wild-type strains, became sensitive to complement-mediated killing in presence of normal human serum. Conversely, naturally serum sensitive strains that did not display a significant polyP pseudo-capsule became resistant to complement in the presence of exogenous polyP. The presence of polyP pseudo-capsule was also critical in the protection from antibacterial activity of cationic antimicrobial peptide, such as cathelicidin LL-37. Results showed that the minimum bactericidal concentration was lower in strains lacking polyP than in those harboring the pseudo-capsule. Data referring to phagocytic killing resistance, assessed by using neutrophil-like cells, showed a significant decrease in viability of mutants lacking polyP on their cell surface in comparison to the wild-type strain. The addition of exogenous polyP overturned the killing phenotype of sensitive strains suggesting that gonococcus could exploit environmental polyP to survive to complement-mediated, cathelicidin and intracellular killing. Taken together, data presented here indicate an essential role of the polyP pseudo-capsule in the gonococcal pathogenesis, opening new perspective on gonococcal biology and more effective treatments.

## Author summary

Innate immunity plays a key role in the clearance of *Neisseria gonorrhoeae*. However, the complex molecular mechanisms evolved by *N*. *gonorrhoeae* to evade the host immune system are still an open-ended question. Moreover, the antibiotic resistance crisis highlights its public health relevance thriving for a deeper understanding of gonococcal pathogenesis to advance our chance to defeat gonococcal infections. Polyphosphate (polyP) is an anionic polymer that *N*. *gonorrhoeae* accumulates both internally and externally to the bacterial cell but its role is still controversial. Here, we demonstrate the presence of a surface exposed pseudo-capsule, made of polyP that shields *N*. *gonorrhoeae* from strategies put in place by the innate immune response, including complement-mediated bactericidal activity of normal human serum, antibacterial activity of cationic antimicrobial peptides, such as cathelicidin LL-37 and the phagocyte-dependent intracellular killing. Our findings highlight an additional strategy used by *N*. *gonorrhoeae* to counteract the host immune system and pave the way to novel approaches to tackle gonococcal infections.

## Introduction

The rising emergency of antibiotic resistance and the lack of alternative treatments make of gonorrhea one of the major global health concerns with an incidence of over 87 million cases per year [1]. The complexity of the gonococcal infection occurring differently in the male and female genital tracts [2], along with a plethora of immune evasion mechanisms, impairing host defenses and preventing bacterial clearance, hinder the development of effective vaccine and new therapeutic approaches. It therefore becomes increasingly important to deeply investigate gonococcal pathogenesis since, despite significant efforts, this complex process is still characterized by unclarified mechanisms [3–6]. Gonococcus is described as unencapsulated bacteria, but its close relation with *Neisseria meningitidis* has often suggested the existence of a polysaccharide capsule. However, the absence of the region A in the capsule cluster of *N*. *gonorrhoeae*, responsible for the polysaccharide synthesis, precludes such possibility [7]. To this purpose, several groups attempted to demonstrate the evidence of capsular material through electron microscopy, producing inconsistent results [7–12]. Noegel and Gotschlich in 1983, isolated high molecular weight polyphosphate (polyP) located outside the cytoplasmic membrane of *N*. *gonorrhoeae* and proposed a possible role as capsule-like material [13]. However due the lack of proper techniques and controls, the existence of a capsule-like material in gonococcus remained controversial [13] and no further research to demonstrate the role of external polyP as pseudo-capsule in gonococcus was described in literature so far. PolyP is a polymer formed by hundreds of thousands of orthophosphates residues, linked by high-energy phosphoanhydride bonds and negatively charged at neutral pH [14]. PolyP has been found to be ubiquitous in all living cells, from bacteria to higher eukaryotes [15–18], occurring in subcellular metachromatic organelles known as volutin and playing an important role in the energy storage [19]. PolyP metabolism is regulated by different enzymes. In particular, its biosynthesis is primarily mediated by the polyphosphate kinase (PPK1), catalyzing the transfer of the γ-phosphate group of ATP into a growing polyP chain [20]. An additional polyphosphate kinase (PPK2), has been reported in some bacterial species, including gonococcus [21]. PPK2 differs from PPK1 because it preferentially uses GTP and Mn^2+^, rather than ATP and Mg^2+^, to catalyze the polymerization of polyP [22]. The catabolic reactions involving polyP are performed by both the endo-polyphosphatase and the exo-polyphosphatase (PPX) [23]. Despite polyP has been described to act as an energy storage polymer [24], numerous findings pointed out the importance of polyP also in biofilm formation, motility, sporulation, surface attachment, stationary phase and stress survival, dormancy and other features linked to the virulence of pathogenic bacteria like *Pseudomonas aeruginosa*, *Campylobacter jejuni* and *Helicobacter pylori*. [16,17,25–29]. Moreover, in *Neisseria meningitidis* polyP has been proposed to be involved in the resistance to the bactericidal activity of normal human serum (NHS) [27,30].

In this study we unravel the existence of a polyP pseudo-capsule in *N*. *gonorrhoeae* and propose its role in gonococcal pathogenesis, specifically in the resistance to killing mediated by human complement, antimicrobial peptides and phagocytosis.

## Results

### Transmission electron and confocal microscopy reveal the presence of a polyP pseudo-capsule in gonococcus

**Fig 1:**
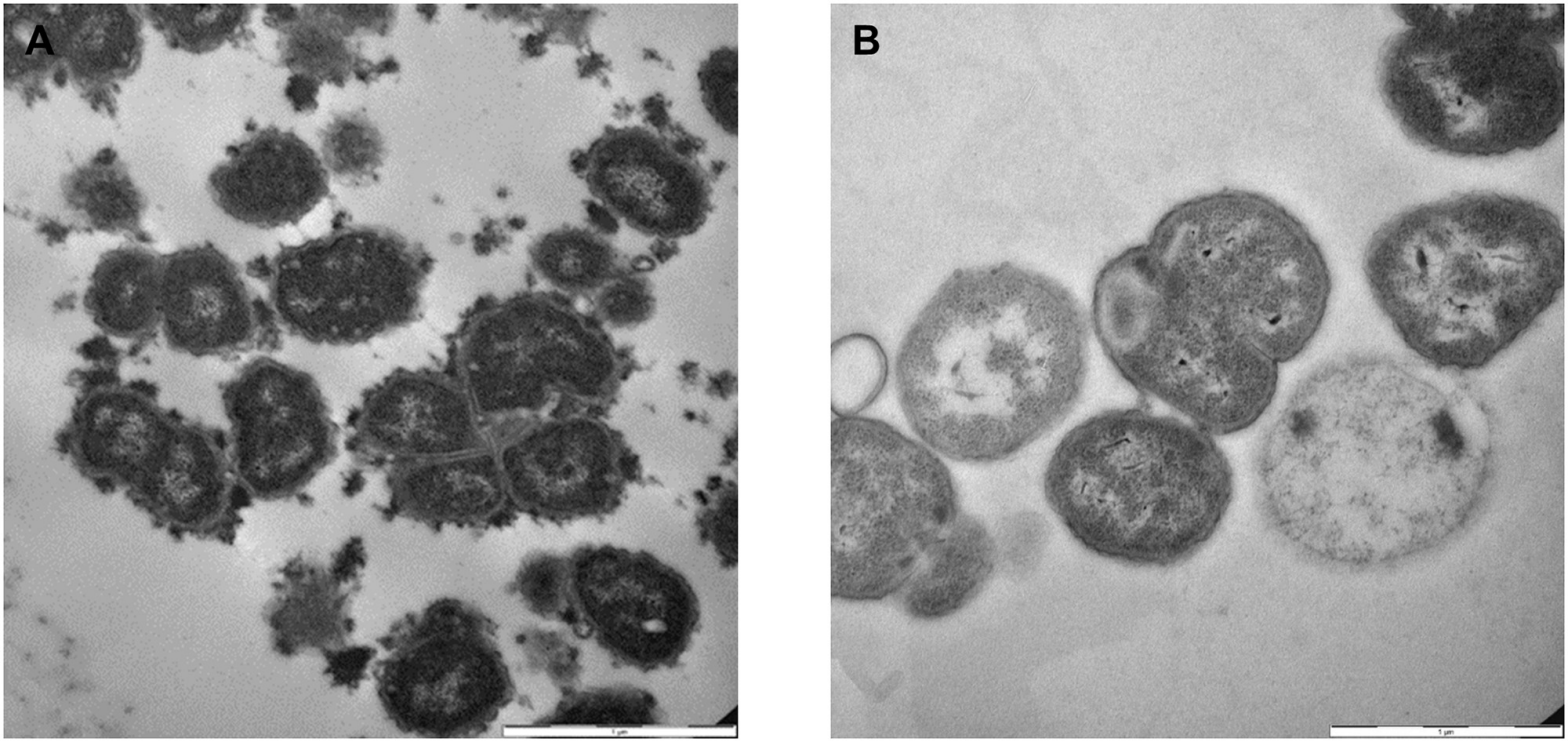
TEM images showing labile negative charged material on *N*. *gonorrhoeae* FA1090. **A**. Cells from *N*. *gonorrhoeae* FA1090 strain treated with Alcian Blue–diamine L-lysine acetate method, show an easily visible layer of capsule-like material. **B**. The staining of *N*. *gonorrhoeae* FA1090 with Alcian Blue only, does not retain extracellular material on the bacterial cell surface. Scale bar = 1 μm.

To allow transmission electron microscopy (TEM) analysis on the PorB.1B *Neisseria gonorrhoeae* FA1090 reference strain, the combination of the cationic dye, Alcian Blue, and the diamine L-lysine acetate, a specific fixative for labile material, was used. As shown in Fig 1A, this procedure of bacterial staining revealed the presence of a capsule-like material surrounding bacterial cells. The use of a cationic dye for the staining suggested the presence of negatively charged material, characterized by different levels of thickness and appearing better assembled in specific parts of the membrane. By omitting the fixative, this structure was not preserved, and it could not be visualized on the gonococcal surface (Fig 1B).

**Fig 2:**
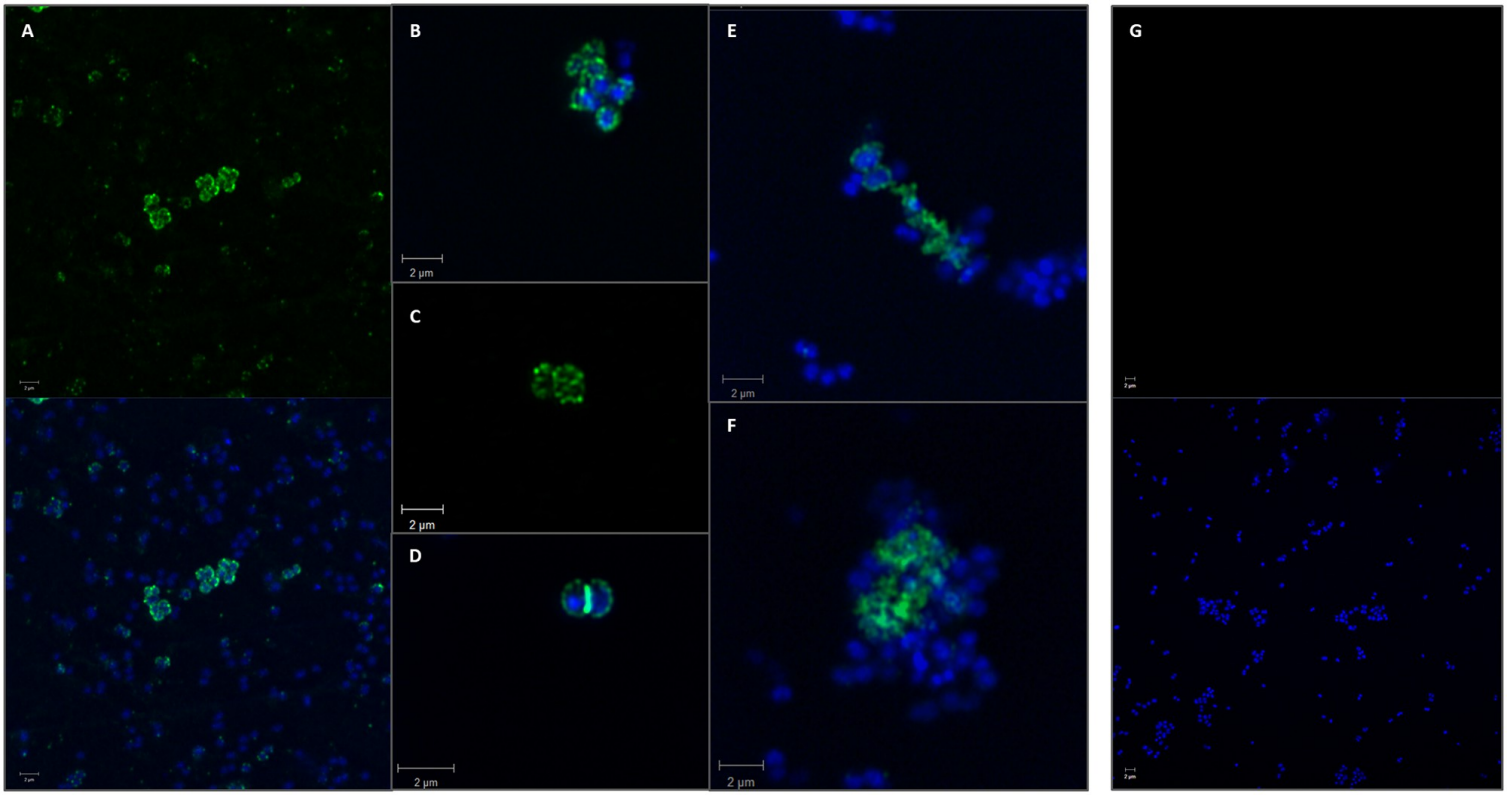
*N*. *gonorrhoeae* FA1090 exposes a polyP pseudo-capsule. Immunofluorescence images show bacterial nucleoid in blue stained with DAPI and *Ec*PPXc binding to polyP in green. Panel **A** shows a bacterial sub-population exposing the polyP pseudo-capsule, panels **B** and **C** exhibit localized polyP spots on the bacterial membrane, panel **D** shows polyP localization on the septum, whereas in the panels **E** and **F**, polyP is organized as a matrix. Panel **G** shows the results of the *Ec*PPXc staining performed in the presence of exogenously added soluble polyP (25 mM). Scale bar = 2 μm.

To investigate the possibility that this capsule-like material could be related to external polyphosphate, the C-terminal polyP binding domain of the *E*. *coli* exopolyphosphatase (*Ec*PPXc), was produced and purified as recombinant His-tagged protein to be used as probe to visualize polyP with confocal microscopy [31]. A clear and strong signal was obtained from the binding of *Ec*PPXc on the surface of *N*. *gonorrhoeae* FA1090, thereby corroborating the hypothesis that the labile negatively charged capsule-like material observed with TEM was composed by polyP (Fig 2A). Localized spots were detected on the surface of a subset of bacterial cells (Fig 2B and 2C) and in some instances a marked high density signal on the septum ring was also seen (Fig 2D). Furthermore, the analysis highlighted the presence of a polyP matrix embedding bacterial aggregates (Fig 2E and 2F). To further verify the specificity of this staining, free soluble polyP was added as a competitor for the binding of *Ec*PPXc. Interestingly, no signal was observed upon competition (Fig 2G) undoubtedly proving that the signal was specifically attributable to polyP.

### The pseudo-capsule is differently allocated on the surface of gonococcal strains

**Fig 3:**
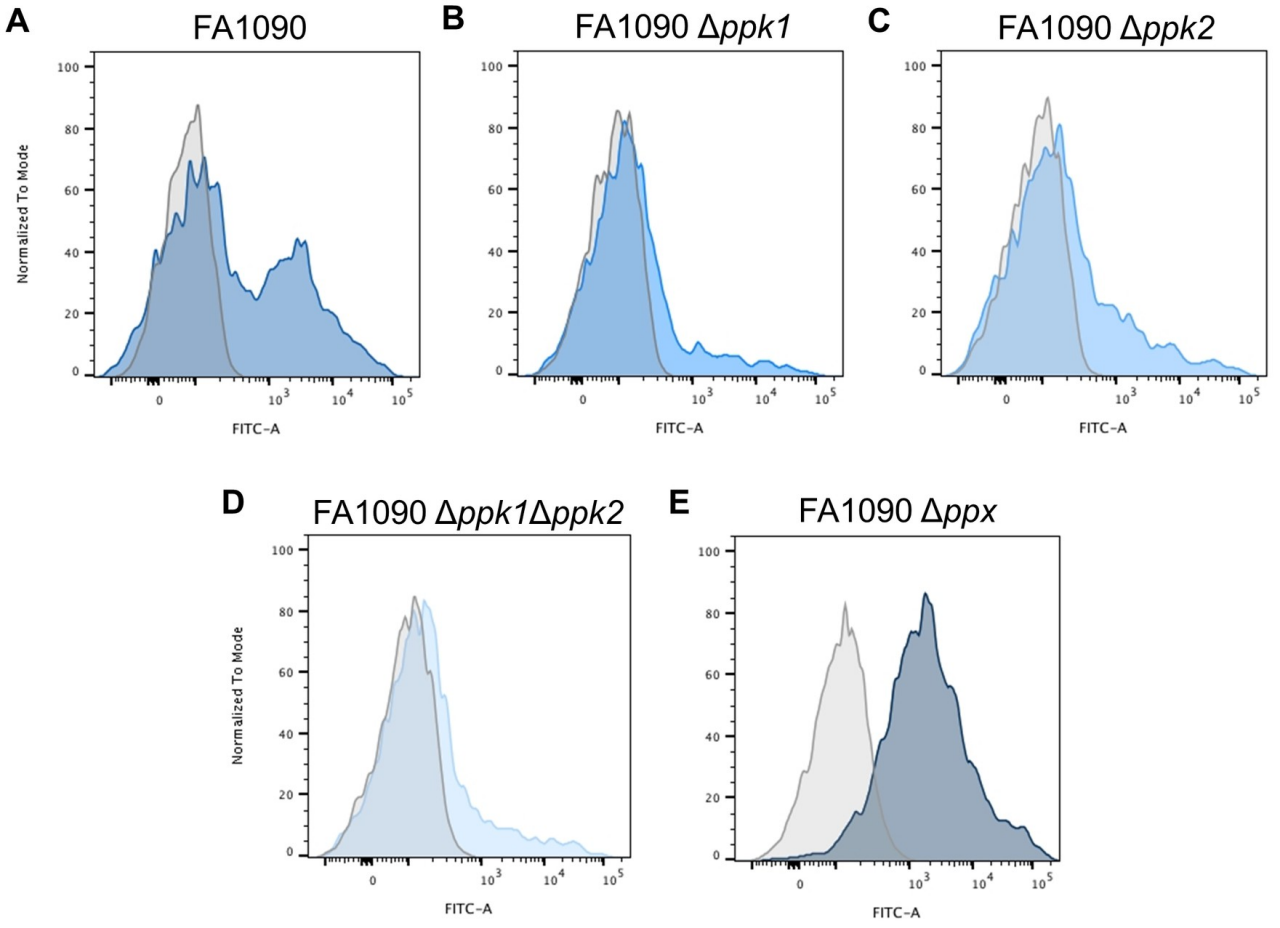
*ppk* and *ppx* deletion mutants differ in surface associated polyP. The presence of polyP pseudo-capsule was assessed by flow cytometry analyses on intact *N*. *gonorrhoeae* FA1090 cells (**A**) and its isogenic mutants FA1090 Δ*ppk1* (**B**), FA1090 Δ*ppk2* (**C**), FA1090 Δ*ppk1*Δ*ppk2* (**D**), FA1090 Δ*ppx* (**E**) after incubation with recombinant His-tagged *Ec*PPXc coupled with anti-His monoclonal antibody combined with 488-conjugated secondary antibody. Blue filled profiles represent stained bacteria. Grey tinted areas indicate strains stained only with secondary antibodies used as negative control.

Results reported in Figs 1 and 2 were also confirmed by flow cytometry analyses performed on *N*. *gonorrhoeae* FA1090 strain. Indeed, results revealed the presence of two distinct subpopulations, one showing a positive fluorescence shift due to the *Ec*PPXc binding to polyP and a second, unreactive to *Ec*PPXc, with no change in fluorescence (Fig 3A). To better evaluate the bacterial response occurring upon alterations of polyP homeostasis, single knockout mutants of each polyphosphate kinase (*ppk*) and exopolyphosphatase (*ppx*) genes as well as double knockout of *ppk1* and *ppk2* genes were generated in FA1090 strain. Flow cytometry analysis indicated that mutants lacking one or both polyP kinases exhibited a strong decrease in the population with surface-associated polyP (Fig 3B, 3C and 3D), whilst the exopolyphosphatase mutant resulted in a single population positive to polyP staining (Fig 3E).

**Fig 4:**
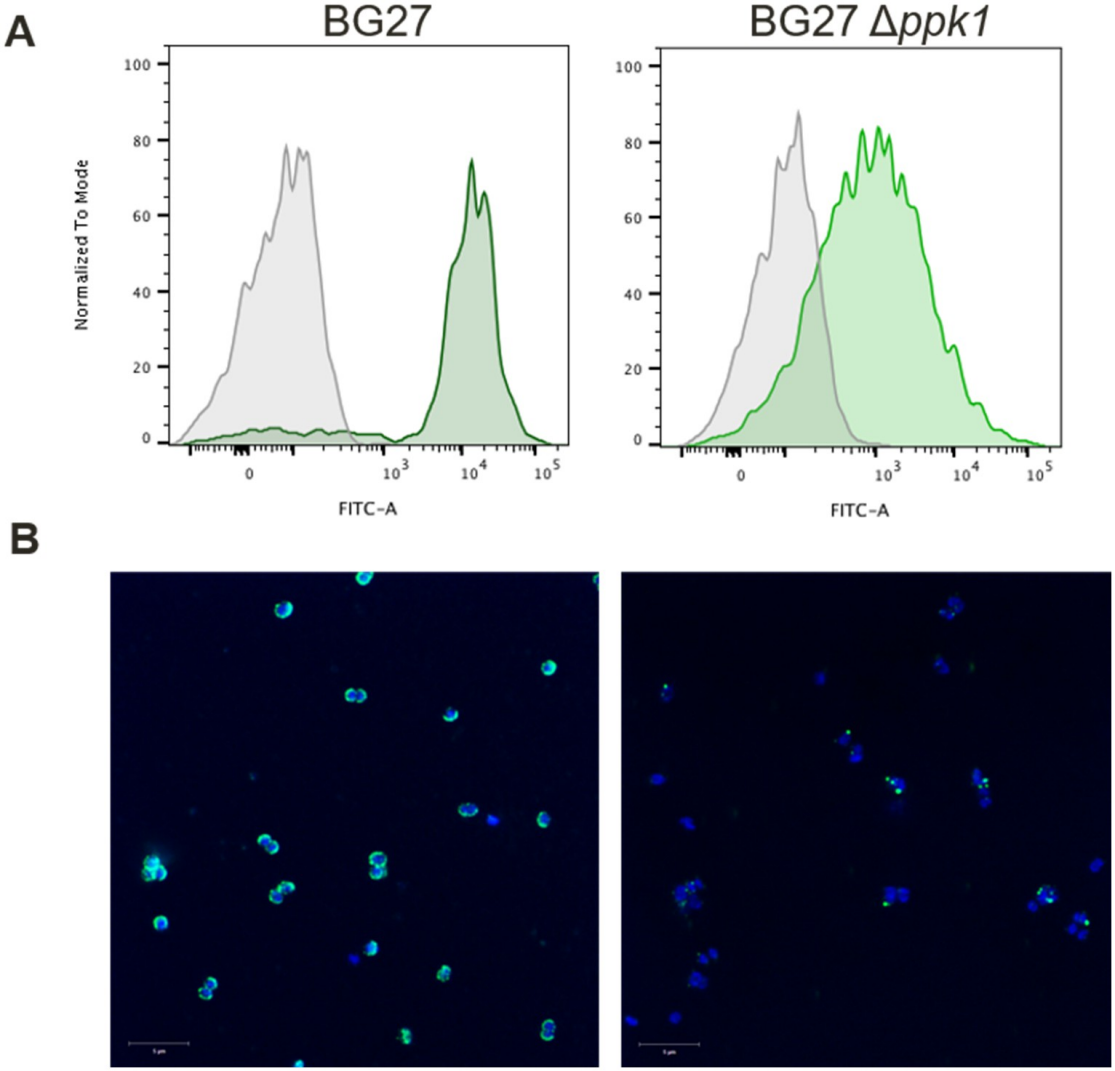
The polyP pseudo-capsule is detected on BG27 clinical isolate and it is impaired by the *ppk1* gene deletion. To visualize the polyP pseudo-capsule, flow cytometry analyses (**A**) and confocal microscopy (**B**) were performed on the clinical isolate BG27 and BG27 Δ*ppk1*. **A**. The grey filled profile represents the negative control stained with the fluorescent secondary antibody only, whereas the green areas represent bacteria with polyP pseudo-capsule, indirectly stained taking advantage of the His-tag of the recombinant *Ec*PPXc. **B**. Immunofluorescence images show bacterial nucleoid in blue stained with DAPI and *Ec*PPXc binding to polyP in green. Scale bar = 5 μm.

To investigate if external polyP was ubiquitously present among different *N*. *gonorrhoeae* strains, the study was extended to low-passaged clinical isolates. To this purpose, the serum resistant *N*. *gonorrhoeae* BG27, isolated in 2013 and expressing the same PorB.1B variant of FA1090 strain, as well as the knockout *ppk1* mutant, were analyzed. Results of flow cytometry (Fig 4A) and confocal microscopy (Fig 4B), revealed the presence of a single positive population characterizing the parental BG27 strain and an overall decrease in surface-associated polyP in the deletion mutant BG27 Δ*ppk1*.

### Surface associated polyP content affects gonococcal complement-mediated serum resistance

**Fig 5:**
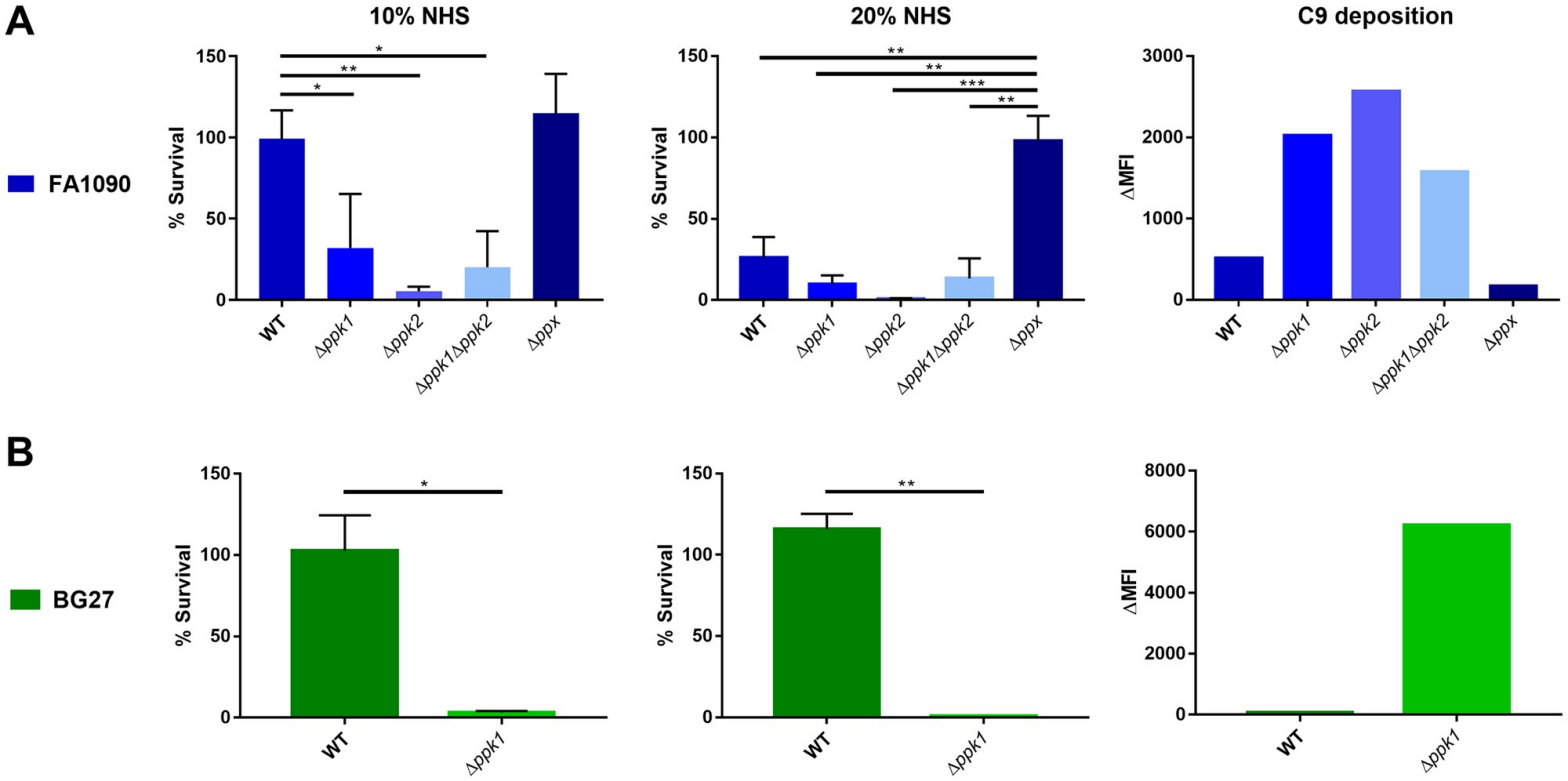
PolyP mutants display different survival rate in NHS, inversely correlating with complement fixation. *N*. *gonorrhoeae* FA1090 (**A**) and BG27 (**B**) were incubated with 10% and 20% NHS. In the case of FA1090 the wild-type strain, the single *Δppk1*, *Δppk2* and the double *Δppk1Δppk2* mutants were tested, while in the case of BG27, the wild-type and the *Δppk1* mutant were used. Results of serum resistance assays are specified as percentage of survival calculated as the ratio of colony forming unit (cfu) after incubation with NHS over cfu incubated with heat inactivated NHS. Data reported are representative of 3 biological replicates performed with 3 different lots of NHS. Standard deviations are represented by vertical bars (*p < 0.05, **p < 0.005, ***p < 0.001). The ability of the strains to trigger complement activation was measured by flow cytometry after incubation of bacteria with 10% NHS. The difference of the median fluorescence intensity (ΔMFI) is calculated by subtracting the MFI in presence of heat-inactivated NHS from the MFI relative to NHS and shows the bacterial population reacting with human anti-C9 antibody. Complement fixation results are representative of one biological replicates performed with one of the NHS lots tested.

To explore whether the polyP pseudo-capsule may be involved in gonococcal pathogenesis and specifically in the evasion from the complement-mediated bactericidal activity of human serum, the parental strain FA1090 and its *ppx* and *ppk* isogenic mutants were incubated with 10% and 20% NHS (Fig 5A). Interestingly, while in the presence of 10% NHS the wild-type strain and *ppx* mutant strains survival was not affected, the three *ppk* mutants were significantly more sensitive to NHS. When the strains were incubated with 20% NHS, the wild-type strain was significantly affected and a further decrease of the survival rate of *ppk* mutants was observed. Conversely, the *ppx* mutant remained still unaltered, even when using this higher concentration of NHS, remarkably representing the most resistant strain. This suggests that the observed sensitivity of FA1090 wild-type to high NHS concentration may be due to the peculiar nature of this strain composed of two sub-populations without and with polyP exposed on their surface (Fig 3A). Based on these results, we could hypothesize a possibly different complement deposition mechanism on the surface of the different strains tested that may be inversely correlated with the polyP content on their surface. In order to support this hypothesis, we analyzed by flow cytometry the deposition of the C9 terminal complement component on the surface of gonococci after incubation with NHS. As expected, deposition of C9 on FA1090 Δ*ppk* mutants was significantly higher when compared with wild-type FA1090 whereas a negligible level of deposition was observed on the surface of FA1090 Δ*ppx* (Fig 5A).

Similar results were obtained when analyzing the survival of BG27 strain to NHS exposure (Fig 5B). As it occurred for FA1090 strain, the survival of BG27 Δ*ppk1* was hampered when using both 10% and 20% NHS. Interestingly, the wild-type BG27 strain was resistant to complement-mediated killing also at the highest serum concentration tested, suggesting that the homogenous presence of polyP in this strain may correlate with a wider protection from complement. These results correlated with the activation of the complement cascade as already shown for FA1090. Indeed, no deposition of C9 was observed for the parental strain, while a significant increase in complement protein levels was detected on the surface of BG27 Δ*ppk1* strain (Fig 5B).

### The serum sensitive F62 strain naturally lacks a polyP pseudo-capsule

**Fig 6:**
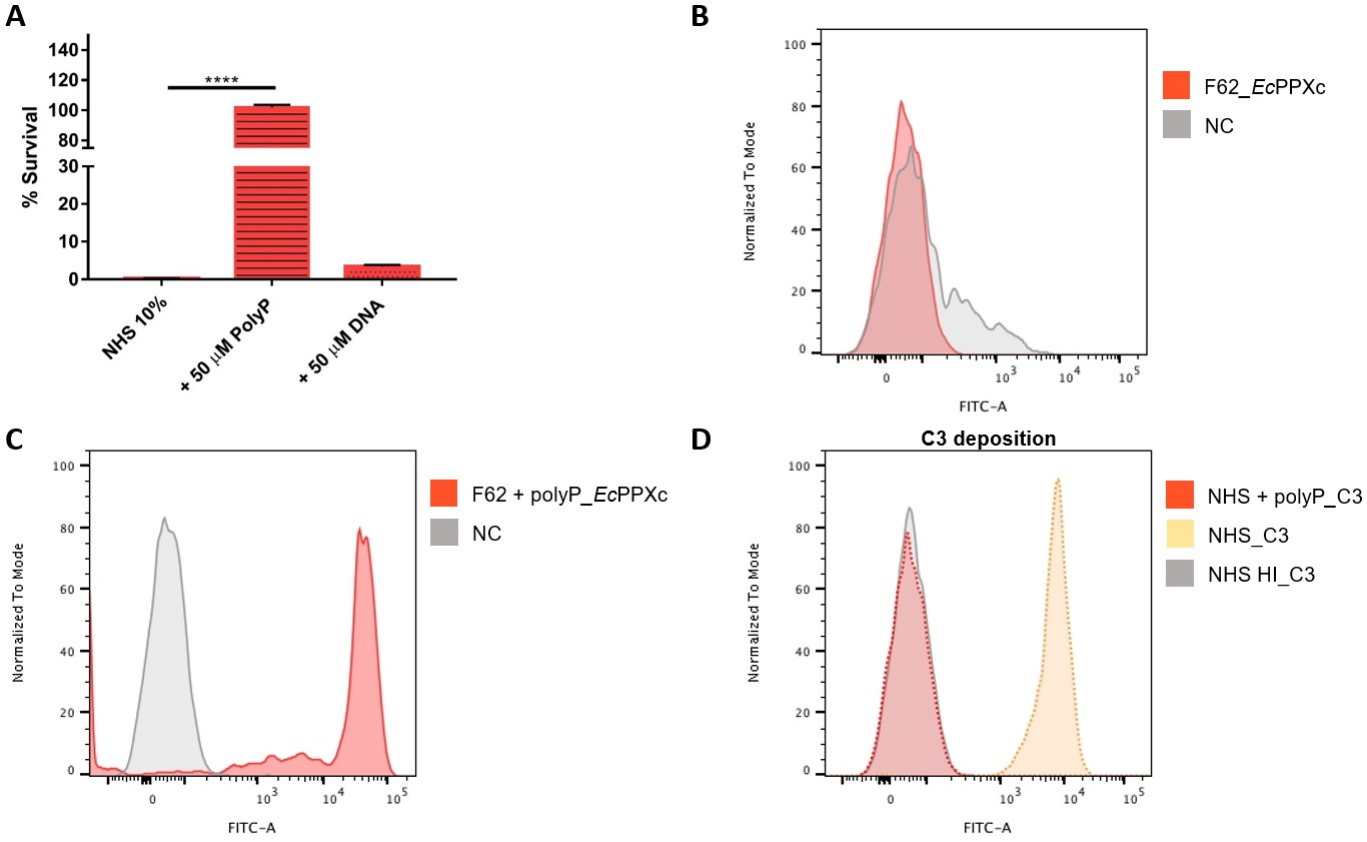
Soluble polyP restores survival in NHS and blocks complement deposition on serum sensitive F62 strain. **A**. Serum resistance of F62 was measured in the presence of 10% NHS. Data are expressed as percentage of survival calculated as the ratio of cfu after incubation with NHS over cfu incubated with heat inactivated NHS. Histograms represent percentage of F62 survival in NHS (filled bar) and in NHS complemented with 50 μM polyP (stripped bar) or with 50 μM DNA (dotted bar). Error bars describe the standard deviation (****p = 0.0001). **B**. F62 polyP pseudo-capsule expression was assessed by flow cytometry through the binding of anti-His antibody targeting the recombinant *Ec*PPXc. The shaded grey profile represents the negative bacterial population, while the red profile shows cells with polyP on their surface. **C**. The ability of soluble polyP to coat F62 surface was evaluated by flow cytometry. Red pic refers to the bacterial population coated by the incubation with 1 mM polyP and recognised by anti-His antibody binding the recombinant *Ec*PPXc, whereas grey pic represents the negative control. **D**. C3 deposition was measured through flow cytometry in the presence of 10% NHS (yellow dotted profile), 10% heat inactivated NHS (grey filled profile), and 10% NHS complemented with soluble 50 μM polyP (red dotted profile). Fluorescence is given by the binding of human anti-C3 fluorescent-labelled antibody.

**Fig 7:**
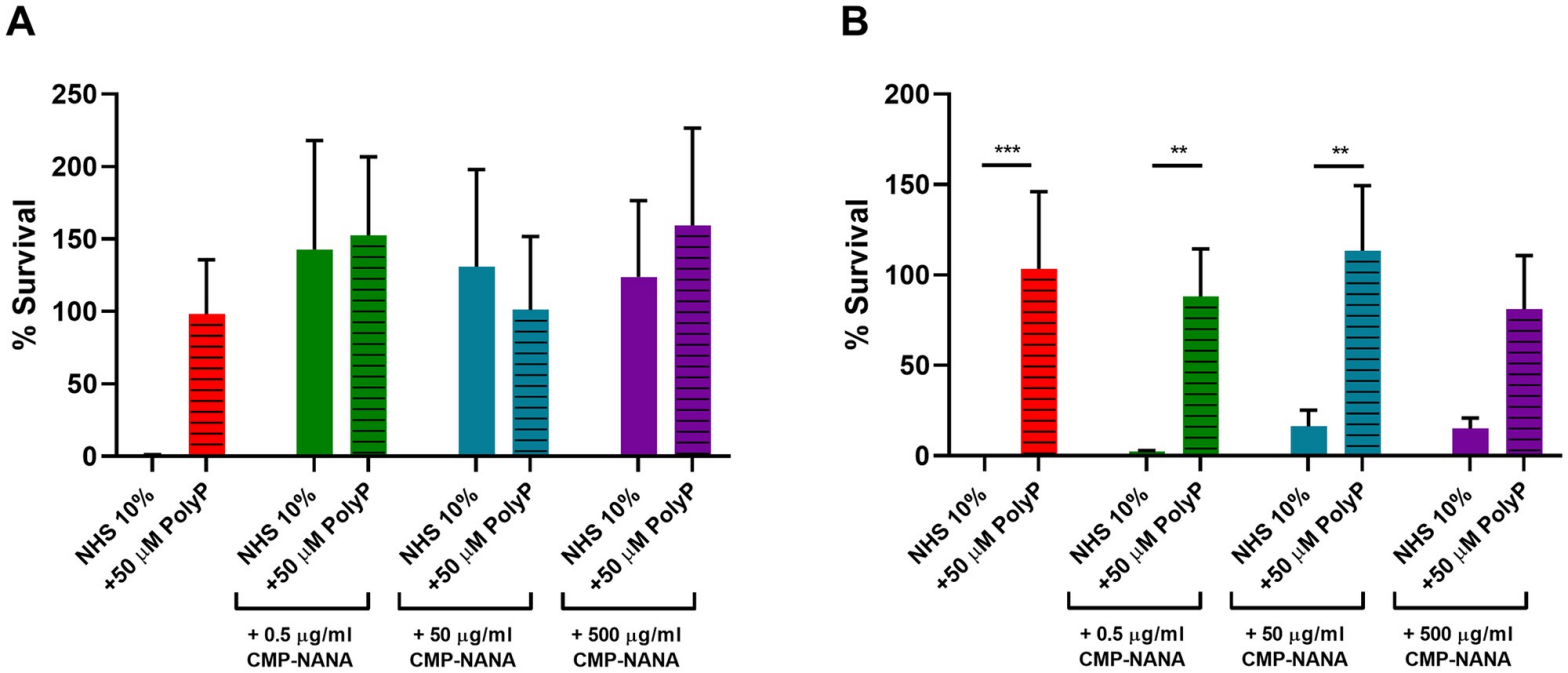
Contribution of polyP in restoring serum resistance of sialylated F62 depends on NHS tested. Serum resistance of F62, grown in the absence or presence of 0.5 μg/mL, 50 μg/mL and 500 μg/mL CMP-NANA (red, green, blue and purple bars, respectively), was measured in the presence of 10% NHS from different donors **A** and **B**. Data are expressed as percentage of survival calculated as the ratio of cfu after incubation with NHS over cfu incubated with heat inactivated NHS. Histograms represent percentage of F62 survival in NHS (filled bar) and in NHS complemented with 50 μM polyP (stripped bar). Error bars describe the standard deviation (**p = 0.005; ***p < 0.0005).

To corroborate the evidence of the role of polyP as one of the mechanisms of gonococcal serum resistance, serum sensitive strains were investigated, specifically the laboratory strain *N*. *gonorrhoeae* F62 [32]. When incubated in the presence of 10% NHS, F62 strain resulted to be sensitive to complement killing (Fig 6A) in agreement with result that no polyP was detected on its surface (Fig 6B). Interestingly, when exogenous free polyP was added to F62 culture, it could be detected by flow cytometry, indicating its binding capability to the bacterial surface (Fig 6C). Given this evidence, we incubated F62 strain with exogenous polyP and tested its capability to survive when exposed to NHS. The results, in line with what observed for strains with naturally surface-exposed polyP, highlighted again the role of polyP as a shield to protect gonococcal surface from complement deposition. Indeed the presence of polyP correlated with F62 survival in NHS (Fig 6A). This ability may be considered polyP-specific and not purely dependent on local electric charge, since the addition of a different negatively-charged molecule like DNA, although coating the bacterial surface (S1 Fig), did not abrogate the NHS killing (Fig 6A). Since gonococci *in vivo* may have a sialylated lipooligosaccharide (LOS) and that the addition of cytidine 5’-monophospho-N-acetylneuraminic acid (CMP-NANA) is able to induce LOS sialylation *in vitro* and increase serum resistance [33,34], we evaluated whether sialylation could interfere with polyP surface association. To this end, F62 strain was grown in the presence of increasing concentration of CMP-NANA (from 0.5 μg/mL to 500 μg/mL) and tested for serum resistance in the presence of NHS from two different donors. Results indicated that LOS sialylation increased survival to different extents, depending on the complement source used (Fig 7). Results depicted in Fig 7A indicate that either CMP-NANA or polyP availability can confer full serum resistance to the tested strain. Consequently, no significant improvement of survival rate was measured testing polyP in combination with CMP-NANA. Noticeably, using a different complement source, CMP-NANA only partially improved F62 survival also at the highest concentration tested, while the addition of exogenous polyP totally restored serum resistance in both sialylated and not-sialylated bacteria (Fig 7B). The transition from a serum sensitive to a serum resistant phenotype in the presence of exogenous polyP may be explained by the ability of polyP to hinder complement cascade activation as shown in Fig 6D, where it is demonstrated that the addition of exogenous polyP abrogated C3 deposition on F62 surface after exposure to NHS.

### PolyP pseudo-capsule confers resistance to cationic antimicrobial peptide

**Fig 8:**
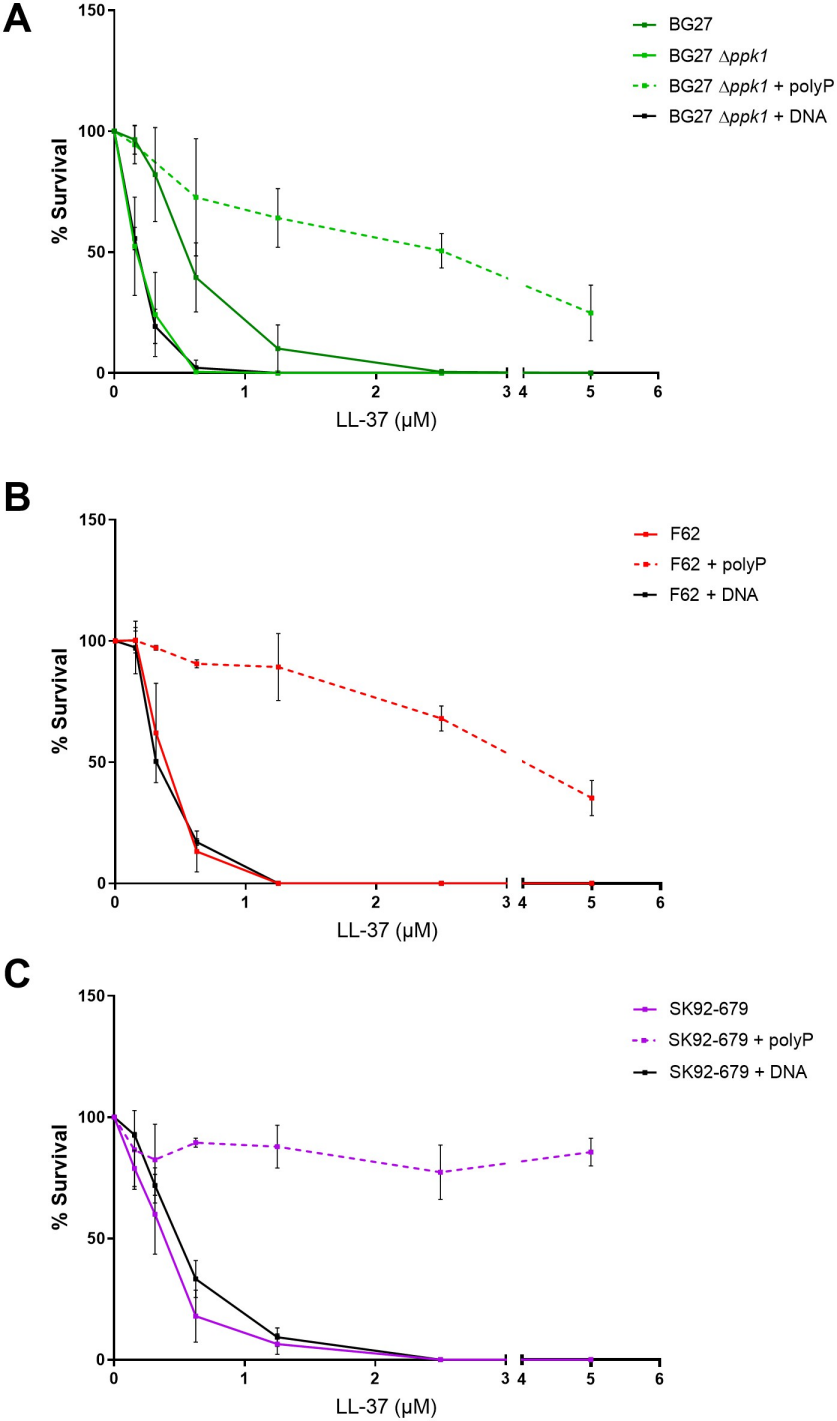
PolyP pseudo-capsule protects *N*. *gonorrhoeae* from antibacterial activity of cathelicidin LL-37. The antibacterial activity of LL-37 was assessed with a cfu counting assay, in which 10^5^ cfu/mL of BG27 and BG27 Δ*ppk1* (**A**), F62 (**B**) and SK92-679 (**C**) were incubated with increasing concentration of the peptide for 1 h at 37°C. The ability of the polyP to restore survival was evaluated by including in the bacterial suspension 20 μM polyP (broken line) or 20 μM DNA (black line). Surviving bacteria were established by plating serial dilution of incubated bacterial suspensions and compared to the number of bacteria present in the control without the peptide. Data are expressed as the mean ± standard deviation of three different experiments.

Given the evidence suggesting a protective role of the gonococcal polyP pseudo-capsule against complement deposition, we questioned whether it could also act as a defense mechanism against antimicrobial peptides. To address this question, we evaluated the bactericidal activity of cathelicidin LL-37 against both the clinical isolate BG27 and its isogenic mutant Δ*ppk1*, previously characterized to have a reduced level of polyP surface exposure (Fig 4). Data revealed (Fig 8A) that the minimum bactericidal concentration (MBC) of LL-37 was four times higher for the parental strain compared to the isogenic Δ*ppk1* mutant (Fig 8A, 2.5 μM vs 0.625 μM, respectively). The addition of exogenous polyP remarkably reduced the LL-37 sensitivity of the mutant strain, with an MBC above the studied concentration range. On the contrary, when BG27 Δ*ppk1* strain was pre-incubated with exogenous DNA, as a negatively charged molecule to mimic the potential charge effect associated with polyP, no effect on bacterial survival was measured, confirming that the protection observed was specifically linked to the presence of polyP rather than to its negative charges.

This analysis was extended to the polyP-negative PorB.1B F62 and PorB.1A SK92-679 strains (Fig 8B and 8C). Both strains displayed the same profile after incubation with increasing concentration of LL-37, showing an MBC of 1.25 μM. Hence, data depicted in Fig 8, likely suggested a direct implication of the polyP in the prevention of LL-37 mediated bactericidal activity as confirmed by experiments where exogenous polyP was included. Indeed, killing of SK92-679 strain even at the highest concentration tested, was totally abolished after 1 h of incubation, instead for F62 strain a slight decrease of survival was already observable with 2.5 μM of LL-37. Resistance of F62 to LL-37 was also tested after growth in the presence of CMP-NANA, the LOS sialylation agent conferring resistance to cationic antimicrobial peptide [35]. The major contribution of polyP than that afforded by sialylation in the protection from the cathelicidin LL-37 was clearly found and was even more evident under stressed conditions such as an increased incubation time of 2 h (S2 Fig).

Gonococcus survival in the presence of defensins was also investigated. In particular, we tested gonococcal susceptibility to α-defensins 1, also known as Human Neutrophil Peptide (HNP-1), mainly present in neutrophil granules. The HNP-1 bactericidal activity was evaluated on both the clinical isolate BG27 and its polyP deficient mutant, resulting in no impairment of the gonococcal survival at each concentration tested (S3A Fig). When testing the activity of another defensin, Human β-Defensin 1 (HBD-1), gonococcal survival was affected either in presence or absence of polyP pseudo-capsule, indicating no involvement of the polyP in the resistance towards this antimicrobial peptide (S3B Fig).

### The polyP pseudo-capsule protects *N*. *gonorrhoeae* from HL60-mediated killing

**Fig 9:**
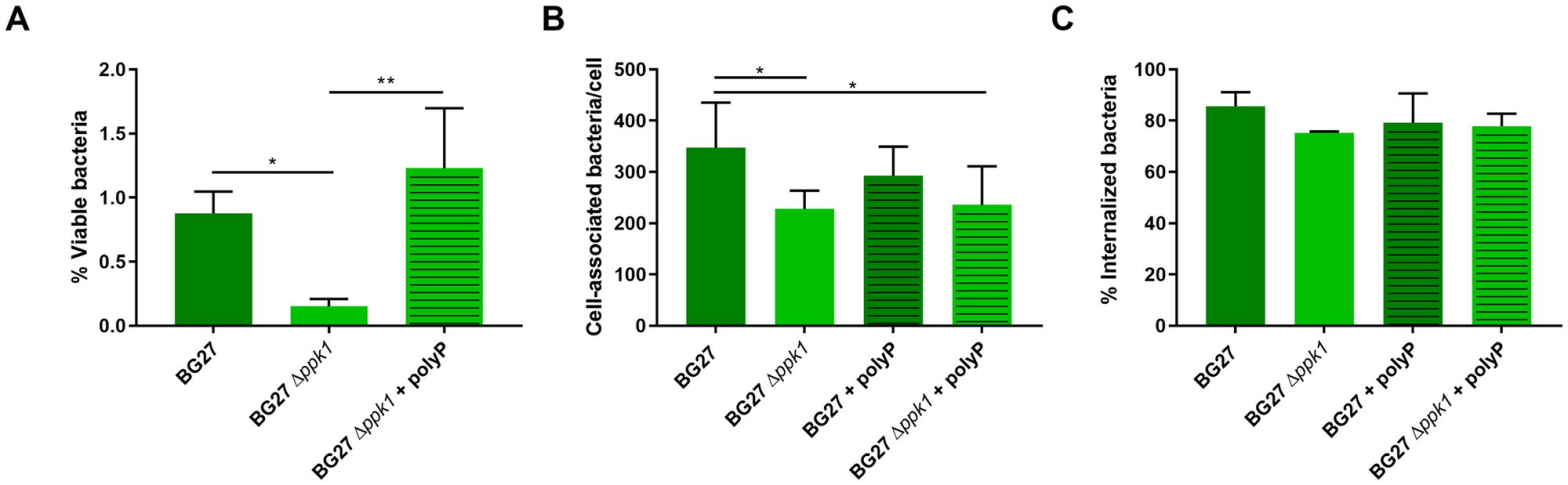
The polyP pseudo-capsule potentiates the survival of engulfed *N*. *gonorrhoeae* by dHL60 and increases bacterial association. **A**. Viability of phagocytized bacteria was assessed by cfu count assay in which dHL60 cells were previously incubated with *N*. *gonorrhoeae* BG27 and BG27 *Δppk1* at multiplicity of infection (MOI) 50. Data are expressed as the percentage of viable bacteria in respect to total infecting bacteria, in the absence or presence of 50 μM polyP, after 1 h of infection. Vertical bars show the standard deviation (*p < 0.05, **p < 0.005). Cell-associated fluorescence volume (**B**) and percentage of internalized bacteria (**C**) were assessed through a fluorescent-based phagocytic assay in which dHL60 were infected at MOI 10 for 1 h with 488 –labelled BG27 and BG27 *Δppk1*, both in the absence or presence of 50 μM polyP. Cell-associated fluorescence volume was calculated using the 488 channel, that was used as value of adherence. The percentage of internalized bacteria (**C**) was calculated subtracting the extracellular fluorescence volume associated to the 546 channel (b) to the cell-associated fluorescence volume (a), according to the formula (a-b) *100/a (*p < 0.05).

The potential involvement of the polyP pseudo-capsule in the gonococcal interaction with neutrophil-like cells was also evaluated. For this purpose, differentiated HL60 cells (dHL60) were infected with the *N*. *gonorrhoeae* clinical isolate BG27 and the isogenic mutant BG27 Δ*ppk1* for 1 h. After previous removal of un-ingested bacteria, dHL60 were lysed and released bacteria were plated and then counted. Results suggested that the viability of the parental BG27 strain was significantly higher than that of BG27 Δ*ppk1* mutant. Interestingly, the viability of the deletion mutant phenotype was reverted after free soluble polyP addition to the reaction. This suggests the involvement of the polyP pseudo-capsule in the increased survival of phagocytized bacteria (Fig 9A). To better understand if this phenotype was linked to a lower degree of bacteria internalization or to a decreased survival capability within dHL60, we developed a methodology to measure the amount of phagocytized bacteria by using an Opera Phenix system. Specifically, after infection for 1 h of dHL60 with 488-labelled bacteria, an anti-gonococcal primary antibody coupled with an AlexaFluor546 secondary antibody was used. This allowed to distinguish between internalized (488-positive only) or cell-associated (488/546-double positive) bacteria. As shown in Fig 9B, despite the number of dHL60-associated bacteria was slightly higher for the parental strain compared to the BG27 Δ*ppk1* mutant, the addition of exogenous polyP did not change the outcome. Furthermore, the percentage of internalized bacteria was comparable among the wild-type BG27, the BG27 Δ*ppk1* mutant strain and the condition encompassing the exogenous polyP addition (Fig 9C). Altogether these results pointed out a major involvement of polyP in the protection from intracellular killing by dHL60, suggesting that the polyP capsule could hinder the ability of phagocytes to kill internalized *N*. *gonorrhoeae*.

## Discussion

PolyP is present in all living cells of both prokaryotes and eukaryotes where it displays multiple functions, including energy storage and metal ion chelator [24,36–38]. In pathogenic bacteria, polyP may also act as a virulence factor, contributing to motility, resistance to stress conditions, heat and acid tolerance or colonization [28,39,40]. In some bacterial species, including *N*. *gonorrhoeae*, polyP may be also localized even on the exterior of the cell. Although data remained still controversial, it has been suggested that its massive extracellular accumulation generated a capsule-like coating [13]. In this study, we demonstrate the existence of a polyP pseudo-capsule in gonococcus and propose its functional role in the evasion from human innate immune system. Utilizing the recombinant C-terminal domain of *E*. *coli* polyP-binding protein (*Ec*PPXc), our experiments provided evidences of a pseudo-capsule and a bacteria-embedding matrix consisting of polyP in the reference strain FA1090. In gonococcus, the metabolism of polyP is mainly regulated by two polyphosphate kinases (PPK1 and PPK2) and one exopolyphosphatase (PPX). As already shown by Tinsley and Gotschlich in 1995, the wild-type FA1090 strain differs from *ppk* mutants in the total amount of polyP accumulation during all growth phases [27]. We here show that these mutations also influence the polyP content located at the exterior of the cell. Indeed, flow cytometry and confocal microscopy analyses indicate: i) the presence of two distinct bacterial sub-populations in the parental strain, one of which exposing polyP on the bacterial surface; ii) the disappearance of this polyP-positive sub-population when one or both *ppk* genes are silenced; iii) and a uniform polyP-positive population associated with the *ppx* deletion mutant. These experimental evidences allowed to exclude a compensatory effect of PPK expression in the Δ*ppx* mutant or of PPX in the Δ*ppk* mutants, as observed in *N*. *meningitidis* by Zhang and colleagues [30]. However, other pleiotropic effects upon these mutations may not be excluded. The biological relevance of the presence of a polyP pseudo-capsule is highlighted by analyzing BG27 strain, a low-passaged clinical isolate harboring the PorB.1B, with polyP homogenously present on the surface of the whole population. In this work we show that a sustained external polyP content correlates with serum resistance, given that gonococcal mutants in which *ppk* genes have been inactivated are highly sensitive to complement-mediated lysis, whereas bacteria lacking PPX and presenting polyP on the external surface, exhibit a resistant phenotype. It is worth underlining that the aforementioned results with the clinical isolate BG27, indicating a resistant phenotype, clearly mirrored those obtained with FA1090 Δ*ppx*, suggesting that in the BG27 strain the amount of surface-associated polyP is naturally higher than that of FA1090 wild-typ*e* strain. To gain further insights into the basis of polyP-mediated serum resistance, we examined the deposition of C9 on bacteria following incubation with human complement. Complement deposition was tested in the presence of 10% NHS since it has been reported that complement factors account for a total amount of 11,5% of undiluted human serum in human cervical mucus [41]. In these experiments, we found an inverse correlation between polyP surface-exposure and complement deposition, strongly suggesting that external polyP may act as a physical-chemical barrier against the activation of the complement cascade on the bacterial membrane. Zhang and colleagues, whose findings on *N*. *meningitidis* are consistent with those observed in this study, proposed a major involvement of polyP in the regulation of the complement alternative cascade since the inhibition of the classical and lectin pathways still correlates with a significant survival advantage for the *ppx* mutant compared to the wild-type [30]. It is acknowledged that antibodies against *N*. *gonorrhoeae*, such as IgG and IgM elicited against PorB and LOS respectively, are present in serum of individuals with no history of gonococcal infections [42,43]. Since we did not remove immunoglobulins from sera used to assess survival and complement deposition on the different strains used in this study, we cannot completely exclude any additional role of polyP in the interference with the classical pathway. However, our studies on FA1090 and BG27 strains suggest a new mechanism used by *N*. *gonorrhoeae* to evade human serum bactericidal activity, an aspect that to date has not been fully elucidated, given that some strains harboring PorB.1B have a serum resistant phenotype in the absence of the binding ability towards both factor H and C4b-binding protein (C4bp) [4,44]. Results obtained using PorB.1B serum sensitive strain F62 seem to corroborate this newly proposed resistance mechanism for gonococcus. Although the unstable serum resistance that characterizes F62 may be ascribed to the inability of this strain to bind C4bp through its porin [44], the absence of a polyP pseudo-capsule shown in the present study introduces a new possible explanation for its *in vitro* sensitivity. The fact that exogenous polyP can be used to coat bacteria that do not naturally produce a pseudo-capsule, suggests that gonococcus might exploit free polyP released by human tissues to escape host complement-mediated killing mechanisms [30,45]. The observed survival rate of F62 strain in normal human serum adjunct of exogenous polyP corroborates this hypothesis, that is further complemented by the negligible C3 deposition on bacterial surface in the presence of externally added polyP. This hypothesis is also in accordance with *in vivo* gonococcal sialylation [46] as our results point up the contribution of polyP pseudo-capsule in the protection from bactericidal activity of normal human serum from different sources also in conditions promoting LOS sialylation. To further explore the polyP-dependent serum resistance the analysis was extended to PorB.1A bearing gonococci. In our experimental settings polyP pseudo-capsule was not detectable on the surface of the laboratory strain SK92-679 and of the clinical isolates WHO-N and WHO-F (S4 Fig). The mechanisms through which PorB.1A strains resist the complement-mediated killing are extensively described in literature and involve the ability of porin to bind factor H and C4bp, thus inhibiting the alternative and classical pathway, respectively [4,44,47,48]. To support this hypothesis, we showed that the addition of exogenous polyP does not give a survival advantage to the gonococci (S5 Fig) since resistance to high concentration of NHS is already conferred by porin.

The idea of a polyP pseudo-capsule as a protective shield led us to investigate its potential involvement in the protection from cationic antimicrobial peptides, another effective mechanism of human mucosal immune response against bacterial pathogens. In particular, LL-37, present in neutrophil granules and secreted by epithelial cells, exerts a potent bactericidal activity against gonococcus [49]. In the present study we show that LL-37 lethality increases with a reduced polyP pseudo-capsule production independently of LOS sialylation, whereas it is attenuated or even abrogated after the addition of exogenous polyP. Interestingly, LL-37 peptide can be found on mucosal sites at a concentration of about 0.44 μM [50] similarly to the concentration range where bactericidal activity against polyP negative strains was observed, highlighting the *in vivo* relevance of polyP-dependent defense mechanism against LL-37.

Another crucial aspect in gonococcal pathogenesis is the interaction with human polymorphonuclear leukocytes (PMNs) within which the bacterium is able to survive and replicate [3,6]. The mechanism by which gonococcus survives PMNs challenge still remains an overarching question. In this study, we provide evidences that polyP seems not to be directly involved in the phagocytic process but it is able to confer protection to intracellular killing by neutrophil-like HL60 cells, a validated cell culture model for *N*. *gonorrhoeae*–neutrophils interaction [51]. BG27 Δ*ppk1* mutant, lacking a polyP pseudo-capsule has a significantly reduced survival in differentiated HL60 cells compared to its parental strain, results that are in agreement with the study of Sureka and colleagues on *Mycobacterium tuberculosis*, in which *ppk1* attenuation compromised the ability to survive in macrophages [52]. Nevertheless, how polyP protects from killing in the aforementioned evasion mechanisms remained unclear. Recently, Rijal and co-workers proposed that polyP secreted by *Mycobacterium smegmatis* can act as a molecular signal to inhibit phagosome acidification and lysosome activity in macrophages to potentiate viability of ingested bacteria, suggesting a possible explanation for the decreased pathogenicity of the bacteria lacking polyP pseudo-capsule [53].

In conclusion, results of this study provide clear evidence that the presence of a polyP pseudo-capsule in *N*. *gonorrhoeae* confers protection against different human immune response mechanisms. However, the different phenotypes observed using different gonococcal strains cannot be explained by the presence of non-functional catalytic sites (S6 Fig), or relevant differences in the expression levels of the enzymes involved in polyP turnover (S7 Fig). These findings were not surprising, since polyP can first function in gonococcus as an energy storage pool. Other gene products may be possibly involved in polyP translocation across the outer membrane and may be differentially regulated in different strains. A multi-omic approach would be beneficial to better clarify the mechanism regulating the production of gonococcal polyP pseudo-capsule. Although further studies are needed, our results introduce a further strategy applied by gonococcus to evade the innate immune response of the host, representing a breakthrough in the field of gonococcal biology and paving the way to a new perspective for prevention strategies and treatments, as well as for new research on gonococcal patho-biological mechanisms [54].

## Materials and methods

### Ethics statement

Human complement source used in the study was obtained according to Good Clinical Practice in accordance with the declaration of Helsinki. Patients have given their written consent for the use of samples of study MENB REC 2ND GEN-074 (V72_92). The study was approved by the Western Institutional Review Board (WIRB).

### Bacterial strains and growth conditions

*N*. *gonorrhoeae* and *Escherichia coli* strains used in this work are listed in Table 1. *N*. *gonorrhoeae* was grown in gonococcal base (GC) agar plates containing 1% v/v Isovitalex at 37°C in an atmosphere of 5% CO_2_. For liquid cultures, bacteria grown for at least 18 h on the plates, were diluted to an OD_600_ of 0.1 in 10 mL of liquid GC—1% v/v Isovitalex or in the chemical defined medium described by Wong and colleagues (WSJM medium) [55] and incubated at 37°C at 160 rpm. To stimulate sialylation of F62, when necessary, the cytidine 5’-monophospho-N-acetylneuraminic acid (CMP-NANA) was added to the liquid medium in a concentration of 0.5 mg/L, 50 mg/L or 500 mg/L. When required, kanamycin (60 mg/L for FA1090 and 80 mg/L for BG27) and chloramphenicol (2 mg/L) were added to culture media at the indicated final concentrations.

*E*. *coli* was grown either in Luria Bertani (LB) liquid medium with shaking at 180 rpm or on LB agar plates overnight at 37°C for plasmid purification or in High Throughput Medium Complex (HTMC) (15 g/L glycerol, 30 g/L yeast extract, 0.5 g/L MgSO_4_, 5 g/L KH_2_PO_4_, 20 g/L K_2_HPO_4_) at 180 rpm at 25°C for protein purification. When required, kanamycin, chloramphenicol or ampicillin were added to achieve a final concentration of 30 mg/L, 20mg/L and 100 mg/L respectively.

**Table 1:** List of strains used in this study.

Name	Genotype	Reference	Year of isolation	Country of isolation
*Neisseria gonorrhoeae* strains				
FA1090	Wild-type PorB.1B	ATCC	1983	USA
FA1090 Δ*ppk1*	Inactivation of *ppk1*	This study	1983	USA
FA1090 Δ*ppk2*	Inactivation of *ppk2*	This study	1983	USA
FA1090 Δ*ppk1*Δ*ppk2*	Inactivation of *ppk1* and *ppk2*	This study	1983	USA
FA1090 Δ*ppx*	Inactivation of *ppx*	This study	1983	USA
BG27	Wild-type PorB.1B	Clinical isolate*	2013	United Kingdom
BG27 Δ*ppk1*	Inactivation of *ppk1*	This study	2013	United Kingdom
F62	Wild-type PorB.1B	ATCC	1960	USA
SK92-679	Wild-type PorB.1A	ATCC	1992	USA
WHO-N	Wild-type PorB.1A	NCTC	2001	Australia
WHO-F	Wild-type PorB.1A	NCTC	1991	Canada
*Escherichia coli* strains				
Mach1	Δ*recA1398 endA1 fhuA* Φ*80*Δ(*lac*)M15 Δ(*lac*)X74 hsdR(r_K_^−^m_K_^+^)	Invitrogen	NA	NA
BL21 (DE3)	F^−^*ompT gal dcm lon hsdS*_*B*_(*r*_*B*_^−^*m*_*B*_^−^) λ(DE3 [*lacI lacUV5*-*T7p07 ind1 sam7 nin5*]) [*malB*^+^]_K-12_(λ^S^)	Invitrogen	NA	NA
DH5-α	F^−^*endA1 glnV44 thi-1 recA1 relA1 gyrA96 deoR nupG purB20* φ80d*lacZ*ΔM15 Δ(*lacZYA-argF*)U169, hsdR17(*r*_*K*_^−^*m*_*K*_^+^), λ^−^	Invitrogen	NA	NA

ATCC, American Type Culture Collection; NCTC, National Collection of Type Cultures; NA, Not Applicable

* Kindly provided by Dr. Derryl Hill, University of Bristol, United Kingdom

### HL60 cell culture and differentiation

The HL60 cell line was obtained from ATCC (American Type Culture Collection). Undifferentiated HL60 cells were cultured in RPMI 1640 with GlutaMAX supplement (Gibco), containing 10% v/v heat-inactivated fetal bovine serum (GE Healthcare) and were kept at concentrations between 10^5^ and 10^6^ cells/mL. Differentiation of HL60 to neutrophil-like cells was achieved by diluting cells to 4 x 10^5^ cells/mL, followed by treatment with 0.78% v/v dimethylformamide (Sigma Aldrich) in culture medium for 4 to 6 days, accordingly to Romero-Steiner et al. [56].

### Construction of plasmids for generation of *N*. *gonorrhoeae* mutants

Genomic DNA and plasmid DNA purification was performed using GenElute Bacterial Genomic DNA kit (Sigma Aldrich) and QIAquick PCR Purification kit (Qiagen) according to the manufacturer’s instruction. Plasmids used in this study are listed in Table 2. To generate the constructs used to knockout the *ppk1* (NGO 0003) and *ppk2* (NGO 2113) genes in *N*. *gonorrhoeae*, Polymerase Incomplete Primer Extension (PIPE) cloning was applied [57]. Briefly, gene loci including the coding sequence and 500 bp upstream and downstream sequences, have been amplified from FA1090 genomic DNA and cloned in modified pET-15 plasmid, using ppkKOupF/ppkKOdoR and ppk2KOupF/ppk2KOdoR, respectively (Table 3). In a second step the coding sequence was replaced by kanamycin and chloramphenicol resistance cassette [57] respectively previously amplified from pET-24 (Novagen) and pSLComCmr [58] with KanKOF/KanKOR and CloKOF/CloKOR primers. To generate construct used to knockout *ppx* (NGO 1041) in *N*. *gonorrhoeae*, the restriction enzymes-based cloning was performed: 500 bp of downstream and 500 bp upstream fragments were individually amplified using primers ppxUPfXBA/ppxUPrsma and ppxDOfsma/ppxDOrXHO and digested with XbaI-SmaI and SmaI-XhoI respectively. In parallel, the chloramphenicol resistance cassette was PCR amplified from pSLComCmr [58] with CloSMAF/CloSMAR and digested with SmaI restriction enzyme. The resulting fragments were ligated into the pBlueScript (Agilent) plasmid previously digested with XhoI and XbaI restriction enzymes. All the PCR amplifications were performed using the KAPA Hi-FI polymerase (KAPA Biosystem). The restriction enzymes digestion was performed following New England Biolabs manual and the ligation with T4 DNA ligase from Thermo Fisher Scientific. DNA samples were analyzed by agarose gel electrophoresis and visualized by staining with SYBR Safe (Thermo Fisher Scientific). *E*. *coli* Mach-1 and DH5-α competent cells were respectively used to perform PIPE and restriction enzyme based cloning steps and transformed by heat shock, according to the Thermo Fisher Scientific protocol. To transform *N*. *gonorrhoeae*, strains were grown on agar plates for at least 18 h. A swab of bacteria was resuspended in PBS, mixed with purified DNA (~1 μg) and then spotted on GC—1% v/v Isovitalex agar plates. The bacteria were then incubated for 5 h in the presence of 5% CO_2_ at 37°C and then finally plated to agar plates containing the appropriate antibiotic. To efficiently screen the transformants, single colonies were expanded onto agar plates in the presence of the appropriate antibiotic and incubated for 18 h at 37°C in an atmosphere of 5% CO_2_. A loopful of bacteria was resuspended in PBS and lysed by incubating the bacterial suspension at 100°C for 10 min in a thermo mixer. After a centrifugation step at 13000 rpm for 5 min, the resulting supernatant containing DNA was used for PCR screening with AccuPrime DNA Polymerase (Thermo Fisher Scientific) according to the manufacturer’s protocol. External oligonucleotides (Table 3) were employed to check if recombinations occurred in the expected genome locus.

**Table 2:** Lists of plasmids used in this study.

Name	Description	Reference
pET-15-*ccdB*	Cloning vector used for generetion of knock-out mutants and for protein expression	Novagene*
pET-15-Δ*ppk1*:KanR	pET-15 with the UP and DOWN *ppk1* regions for the recombination which contains a Kan resistance cassette	This study
pET-24	Plasmid used to amplify the Kan resistance cassette	Novagene
pET-15-Δ*ppk2*:CmR	pET-15 with the UP and DOWN *ppk2* regions for the recombination which contains a Cm resistance cassette	This study
pSLComCmr	Plasmid used to amplify the Cm resistance cassette	[58]
pET-15-*Ec*PPXc	Construct for *Ec*PPXc expression with 6xHis N-terminal tag	This study
pBlueScript (pBS)	Cloning vector	Stratagene
pBS-Δ*ppx*:CmR	pBS with the UP and DOWN *ppk1* regions for the recombination which contains a Cm resistance cassette	This study

KanR, Kanamycin resistance cassette; CmR, chloramphenicol resistance cassette;

* modified from Novagene to adapt to PIPE cloning

**Table 3:** List of oligonucleotides used in this study.

Name	Sequence	Description	Reference
V-pipe UnivF	TAACGCGACTTAATTGGCCAGTGTGCCGGTCTCCG	v-PCR for knock-out mutants (Fw) and protein expression	This study
pET15-KOrv	TCACAATTCCCCTATAGTGAGTC	v-PCR for knock-out mutants (Rew)	This study
petTEVrev	GCCCTGGAAGTACAGGTTTTCGTGATGATGATGATGATGGCTGCTGCCCATGGTATATC	v-PCR for protein expression	This study
ppkKOupF	ATAGGGGAATTGTGAGTCATCGTCGACACATCCAAG	Construction of Δ*ppk1*:*kanR* mutant (Fw)	This study
ppkKOdoR	AATTAAGTCGCGTTAGCTGGAACGTGTGTCCATTC	Construction of Δ*ppk1*:*kanR* mutant (Rew)	This study
ppkKOdoF	GGATCCCCATGGATACCCGCACAAGCACTTCCCCCATAC	Construction of Δ*ppk1*:*kanR* mutant (Fw)	This study
ppkKOupR	TCCTTCAGACGGCATAAGGTTCTCCCAAAGATGG	Construction of Δ*ppk1*:*kanR* mutant (Rew)	This study
KanKOF	ATGCCGTCTGAAGGATCCGACTAACTAGGAGGAATAAATG	i-PCR for KanR insertion (Fw)	This study
KanKOR	TATCCATGGGGATCCTCATTATTCCTTCCAGGTACTAAAAC	i-PCR for KanR insertion (Rew)	This study
ppkKOextF	CAGCCGGATGCGGTCAGTG	Δ*ppk1*:*kanR* screening (Fw)	This study
ppkKOextR	GTGTTGTCCCGTGCCGAAC	Δ*ppk1*:*kanR* screening (Rew)	This study
ppk2KoupF	ATAGGGGAATTGTGATCTAGACTGAATGTCGGGCAAGCCAGC	Construction of Δ*ppk2*:*cmR* mutant (Fw)	This study
ppk2KodoR	AATTAAGTCGCGTTACTCGAGCTTCGAGCCTACCCTGCTGAC	Construction of Δ*ppk2*:*cmR* mutant (Rew)	This study
ppk2KodoF	GGATCCCCATGGATACCCGGGTTGCCGGTGAAGTAATAAAAATGC	Construction of Δ*ppk2*:*cmR* mutant (Fw)	This study
ppk2KoupR	TCCTTCAGACGGCATCCCGGGAAAGGTCTCCTATTGTATTTCAG	Construction of Δ*ppk2*:*cmR* mutant (Rew)	This study
cloKOF	ATGCCGTCTGAAGGATCCGTCAACCGTGATATAGATTGAAAAGTG	i-PCR for CmR insertion (Fw)	This study
cloKOR	TATCCATGGGGATCCGATCCACGCGTCTTAAGGCGG	i-PCR for CmR insertion (Rew)	This study
ppk2KoextF	CGGTCGAGGTAATCGGTTTCG	Δ*ppk2*:*cmR* screening (Fw)	This study
ppk2KoextR	GCCGTCAAACAAGGTGCG	Δ*ppk2*:*cmR* screening (Rew)	This study
ppxUPfXBA	TTATCTAGACCGGGACAATTGTGTACCGCTTC	Construction of Δ*ppx*:*cmR* mutant (Fw)	This study
ppxUPrsma	TAACCCGGGGGCGGATACCGGTGGGGGAAAAACG	Construction of Δ*ppx*:*cmR* mutant (Rew)	This study
ppxDOfsma	AATCCCGGGACCTTGACAAACCAAATGCCGTCTAAATG	Construction of Δ*ppx*:*cmR* mutant (Fw)	This study
ppxDOrXHO	ATTCTCGAGGCAATGATGGCGGCTGCCTTGGCAGC	Construction of Δ*ppx*:*cmR* mutant (Rew)	This study
cloSMAF	ATTCGCCCGGGGTCAACCGTGATATAGATTGAAAAG	CmR amplification with SmaI restriction site (Fw)	This study
cloSMAR	ATTCGCCCGGGACGCGTCTTAAGGCG	CmR amplification with SmaI restriction site (Rew)	This study
ppxKOextF	GACGCTGATGTATTCCGCGC	Δ*ppx*:*cmR* screening (Fw)	This study
ppxKOextR	TATTGATTGCCGCAATGATGG	Δ*ppx*:*cmR* screening (Rew)	This study
Ecppx_F	CTGTACTTCCAGGGCATGGAAGGACGTTTCCGTCATCAG	Construction of pET 15-*Ec*PPXc (Fw)	This study
Ecppx_R	AATTAAGTCGCGTTATTAAGCGGCGATTTCTGGTGTAC	Construction of pET 15-*Ec*PPXc (Rew)	This study
ppkRTfor	CGCGCCCGCTGAACAAATCG	qRT-PCR *ppk1* gene (Fw)	This study
ppkRTrev	AGGATGCGCGGTGCTTGGAC	qRT-PCR *ppk1* gene (Rew)	This study
ppk2Rtfor	CGCCGCCTACGAAGCCG	qRT-PCR *ppk2* gene (Fw)	This study
ppk2Rtrev	GCCCTTACCTGCCGCATC	qRT-PCR *ppk2* gene (Rew)	This study
ppxRTfor	GGGATTTCGCCGTCGGCAC	qRT-PCR *ppx* gene (Fw)	This study
ppxRTrev	GAGGGCGCGCATTCCCTTG	qRT-PCR *ppx* gene (Rew)	This study
ppxWTF	GGCACTGGCAGACAATAC	DNA presence control check in RNA samples (Fw)	This study
16SRTF	GTAGGGTGCGAGCGTTAATC	qRT-PCR *16s* gene (Fw)	[59]
16SRTR	CATCGGTATTCCTCCACATCTC	qRT-PCR *16s* gene (Rew)	[59]

KanR, Kanamycin resistance cassette; CmR, chloramphenicol resistance cassette

### Transmission electron microscopy

*N*. *gonorrhoeae* FA1090 colonies grown overnight at 37°C in the presence of 5% CO_2_ on GC—1% v/v Isovitalex plates, were suspended to 0.1 OD_600_ in 50 mL GC- 1% v/v Isovitalex liquid broth prewarmed to 37°C and incubated until the log phase (0.5 OD_600_). The cell suspension was harvested at 4000 rpm for 5 min. Samples were immersed in 2.5% v/v glutaraldehyde, 2% v/v paraformaldehyde in 0.1 M cacodylate sucrose buffer pH 7.2, containing 0.15% v/v Alcian blue and 1.55% w/v L-lysine acetate, for 20 min at 4°C. This was followed by the primary fixative alone (without L-lysine-acetate), for 3 h at 4°C. For the negative control the L-lysine acetate was not included in the solutions. The fixed cells were then washed in the same buffer (three rinses of min each), and post-fixed with 1% osmium tetroxide in 0.1 M cacodylate buffer containing 0.15% v/v Alcian blue for 1 h at 4°C. Specimens were washed three times, 15 min each, at 4°C in the same buffer, dehydrated in a graded ethanol series, then infiltrated with mixtures of LRWhite resin/ethanol in different percentages. At the end of the procedure, samples were embedded in LRWhite resin, 2 days at 50°C. Blocks were cut with Reichert Ultracut ultramicrotome using a diamond knife. For TEM, Ultrathin sections (60–80 nm) were collected on copper grids, stained with uranyl acetate and lead citrate, and observed with a JEOL 1200 EX II electron microscope. Micrographs were captured by the Olympus SIS VELETA CCD camera equipped with iTEM software.

### Production and purification of a recombinant His-tagged C-terminal domain of the *E*. *coli* exopolyphosphatase

The enzymatically inactive C-terminal domain of the *E*. *coli* strain K12 exopolyphosphatase (*Ec*PPXc), that is a specifically polyP binding domain [31], was amplified and cloned in the pET 15 plasmid. *E*. *coli* K12 genomic DNA was used as DNA template for the *EcPPXc* gene amplification. The PCR product amplified using primers Ecppx_F and Ecppx_F (Table 3) was cloned in a pET 15 plasmid applying a PIPE cloning strategy, thus obtaining the pET 15-*Ec*PPXc. The plasmid was then transformed into *E*. *coli* BL21 (DE3). Single colonies of recombinant *E*. *coli* were inoculated in 5 mL of LB added with 100 mg/L ampicillin and grown overnight at 37°C, 180 rpm. The cultures were then diluted 1:100 in 100 mL of HTMC medium with 100 mg/L ampicillin. Protein expression was induced with isopropyl D-thiogalactoside (IPTG) at a final concentration of 1 mM after approximatively 6 h growth at 25°C, 180 rpm. Then the culture was incubated overnight until stationary phase.

For protein purification, the bacterial pellet was resuspended in the equilibration buffer (Tris-HCl 20 mM pH 8, NaCl 300 mM) and mechanically lysed by sonication (amplitude 65%, 30 sec ON/OFF, 30 min in total). Bacterial debris were removed by centrifugation and the recombinant protein was purified using the N-terminal His-tag on a cobalt-based chelating column (HiTrap TALON crude, GE Healthcare Life Science) by Immobilized Metal Affinity Chromatography (IMAC). The column was washed with equilibration buffer and proteins bound to the resin were then eluted with imidazole gradient (from 0 mM to 300 mM imidazole) in equilibration buffer. After this step, sample purity was assessed on an analytical size-exclusion by Ultra Performance Liquid chromatography (SE-UPLC) using BEH200 4.6 x 300 mm columns (Waters) and using as running buffer 10 mM NaH_2_PO_4_, 400 mM (NH_4_)_2_SO_4_, pH 6.2. Samples were dialyzed in Tris-HCl 20 mM pH 8, NaCl 300 mM. Finally, proteins were analysed by SDS-PAGE under reducing condition followed by Coomassie blue staining (Giotto) (S8 Fig). Protein content was determined using the BCA assay from Thermo Scientific.

### Staining of polyP on bacterial surface by confocal microscopy

*N*. *gonorrhoeae* colonies grown overnight at 37°C in the presence of 5% CO_2_ on GC—1% v/v Isovitalex plates, were suspended to 0.1 OD_600_ in 10 mL GC-1% v/v Isovitalex broth liquid prewarmed to 37°C. Cell suspensions of 0.5 OD_600_ were harvested at 8000 × g for 5 min, washed in sterile PBS at 0.5 OD_600_, resuspended in 250 μL PBS containing 1 ug/mL of *Ec*PPXc and incubated for 30 min at room temperature (RT). For competition experiments, 25 mM polyP (Graham’s salt, Merck) were added. The samples were washed once and fixed with 2% v/v formaldehyde in PBS for 20 min and then 100 μL were spotted on polylysine-coated slide (Thermo Scientific). After washing, cells were incubated for 1 h with 100 μL primary antibody (1:250 monoclonal anti 6x-His Tag antibody, Thermo Fisher Scientific). After three washing steps, 100 μL of secondary antibody was added (30 min, 1:1000 Alexa Fluor 488 goat anti-mouse antibody, Thermo Fisher Scientific). Finally, three final washings were then performed, and labelled preparations were mounted with ProLong Gold antifade reagent containing DAPI (Invitrogen). Microscope slides were then analyzed with a 100x oil objective mounted on a Zeiss LSM-710 confocal microscope and images captured using ZEN Black software (Carl Zeiss). For DNA staining, bacterial suspension directly fixed with 2% v/v formaldehyde was spotted on the microscope slides. Cells were then incubated for 5 min with 50 μM of DNA (deoxyribonucleic acid, low molecular weight from salmon sperm, Sigma Aldrich). After washing, cells were incubated for 1 h with 100 μL primary antibody (1:250 monoclonal anti-ds DNA antibody, Abcam). After three washing steps, 100 μL of secondary antibody was added (30 min, 1:1000 Alexa Fluor 488 goat anti-mouse antibody, Thermo Fisher Scientific). Finally, microscope slides were prepared as described above for their visualizations.

### Staining of polyP on bacterial surface by Fluorescence Activator Cell Sorting (FACS)

*N*. *gonorrhoeae* colonies grown overnight at 37°C in the presence of 5% CO_2_ on GC—1% v/v Isovitalex plates, were suspended to 0.1 OD_600_ in 10 mL GC-1% v/v Isovitalex broth liquid prewarmed to 37°C and incubated until exponential phase at 37°C at 160 rpm. Cell suspensions were sub-cultured into Wong medium and followed until early log phase (0.3–0.4 OD_600_). A volume of bacterial culture was collected to have 1 OD_600_, spun down and fixed for 1 h at RT in 1 mL of 0.5% v/v formaldehyde in PBS. The bacterial pellet was then resuspended for 1 h at RT in blocking buffer (1% w/v BSA in PBS) to reach 0.25 OD_600_. PolyP staining was carried out by incubating for 30 min at RT 50 μL (OD_600_ 0.0125) of the cell suspension with 50 μL of blocking buffer containing the recombinant protein *Ec*PPXc, to reach a final concentration of 100 μg/mL. As a negative control the cell suspension is incubated with the same volume of blocking buffer. The samples were washed once and incubated for 1 h with 100 μL primary antibody (1:250 monoclonal anti 6x-His Tag antibody, Thermo Fisher Scientific). After two washes, 100 μL of FITC-conjugated goat anti-mouse IgG secondary antibody was added at a 1:500 dilution and incubated for 30 min (Sigma Aldrich). After final washings, cells were resuspended in PBS. Bacterial fluorescence was recorded with BD FACS CANTO II (BD Bioscience) and data were analyzed using Flow-Jo v.10 (FloJo, LLC).

### Serum resistance assay

Gonococci were grown to the log phase (OD_600_, between 0.4 and 0.5) in GC-1% v/v Isovitalex medium, subcultured into Wong medium and then followed until early exponential phase (0.3–0.4 OD_600_). Fifty microliters of bacterial suspension, diluted 1:50 in a sterile buffer containing 1% w/v BSA 0,1% w/v glucose in PBS was incubated for 1 h at 37°C in the same volume of 40% v/v and 20% v/v freshly thawed NHS, in order to assess bacteria survival in the presence of 20% v/v and 10% v/v of complement source, respectively. For competition experiment 50 μM of polyP (Graham’s salt, Merck) or 50 μM of DNA (deoxyribonucleic acid, low molecular weight from salmon sperm, Sigma Aldrich) were added to the reaction mixture. Experimental controls included bacteria incubated with heat-inactivated complement at 56°C for 30 min. Aliquots (10 μL) of diluted bacterial suspension were collected at the beginning of the experiment for enumeration of cfu at time zero (T_0_) and after the incubation time to be disseminated into GC-1% v/v Isovitalex agar plates by tilt method. The percentage of surviving bacteria was expressed relatively to that of decomplemented controls, whereas the standard deviation was determined on the ratio of the two means, represented by number of bacteria in the NHS and those in the heat inactivated NHS. For FA1090 panel, statistical significance was calculated by two-way ANOVA for the 10% NHS condition and by ordinary one-way ANOVA for the 20% NHS condition. For BG27 panel, statistical significance was determined by unpaired t-test. For F62 panel, statistical significance was calculated by ordinary one-way ANOVA (*p < 0.05, **p < 0.005, ***p < 0.001, ****p = 0.0001). Human complement source used in the study was obtained according to Good Clinical Practice in accordance with the declaration of Helsinki. Patients gave their written consent for the use of samples of the study MENB REC 2ND GEN-074 (V72_92).

### Complement binding studies

For complement deposition studies, gonococci, grown as described above, were fixed in 0.5% v/v formaldehyde for 1 h, then resuspended in blocking buffer and finally 10^5^ cells were incubated with 10% v/v NHS (or heat-inactivated NHS) for 30 min at 37°C. For competition experiments, 50 μM of polyP (Graham’s salt, Merck) was added to the reaction mixture. After two washes with 100 μL PBS, the samples were incubated for 1 h with 100 μL FITC-conjugated mouse anti human/mouse C3/C3b/iC3b antibody (1:100 dilution; Caderlane) or mouse anti-human C9 monoclonal antibody (at a 1:250 dilution; Thermo Fisher Scientific). Following washing steps, 100 μL of FITC-conjugated goat anti-mouse IgG secondary antibody was added at a 1:500 dilution and incubated for 30 min (Sigma Aldrich). Finally, cells were resuspended in PBS and bacterial fluorescence was recorded with BD FACS CANTO II (BD Bioscience) and then analyzed using Flow-Jo v.10 (FloJo, LLC).

### Phagocytosis assay

Phagocytic uptake was assessed adapting a protocol applied by Chen and Seifert [51]. In a 24-well plate, 10^6^ differentiated HL60 (dHL60) were seeded in a volume of 0.4 mL of infection medium consisting of RPMI 1640 with GlutaMAX supplement and were then allowed to adhere at 37°C and 5% CO_2_ for 30 min. The adherent dHL60 cells were then infected with 5 × 10^7^ cfu of *N*. *gonorrhoeae* BG27 strain and BG27 Δ*ppk1* mutant, grown in GC-1% v/v Isovitalex medium, in 0.1 mL volume of infection medium. In the condition with polyP, exogenous polyP was added to the infection medium to a final concentration of 50 μM polyP (Graham’s salt, Merck). The infections were carried out at 37°C and 5% CO_2_. At 1 h post infection, unbound bacteria were removed through a washing step with PBS. Cells were then lysed in 1% saponin in PBS to determine both adherent and internalized bacteria or were treated with 100 mg/L of gentamycin for 1 h to determine internalized bacteria only. Serial dilutions were then performed and plated on GC-1% v/v Isovitalex agar plates. The experiment was performed in triplicate while the complementation with free polyP in duplicate. Viable internalized bacteria were established as the ratio of phagocytized bacteria to total bacteria (adherent plus internalized bacteria). Statistical significance was determined with a one-way ANOVA, using GraphPad Prism 7 software (*p < 0.05, **p < 0.005).

### Fluorescent-based phagocytosis assay

*N*. *gonorrhoeae* BG27 strain and its Δ*ppk1* mutant were chemically conjugated with Oregon Green 488 (1:200 in PBS, Thermo Fischer Scientific), for 15 min at 37°C. To remove excess of dye, the samples were washed once with PBS and then bacteria were diluted in infection medium at the concentration of interest. Infection was performed as described above. Cells were then fixed using 4% formaldehyde for 30 min and resuspended in blocking buffer (1% w/v BSA in PBS). To distinguish extracellular from internalized bacteria, a mouse anti-whole cell gonococcal serum was used in combination with a goat anti-mouse 568-conjugated secondary antibody (30 min, 1:1000 Alexa Fluor 568 goat anti-mouse antibody, Thermo Fisher Scientific). Finally, samples were stained with markers for cell membrane (1:1000 Cell Mask Deep Red Plasma Membrane, Invitrogen) and nucleic acid (1:1000 4′,6-diamidino-2-phenylindole, DAPI 1 mg/mL, Thermo Fisher Scientific). and transferred to an imaging plate (96 well Clear, Greiner Bio-One). An Opera Phenix Plus instrument (Perkin Elmer) equipped with a 63x water objective was used to analyze the samples. In particular, a total of 25 z-stacks per field of acquisition (0.5 μm distance between planes) and 81 fields per well were acquired in confocal mode. Images were then analyzed in 3D mode by using the Harmony software. In particular, cell nuclei and cytoplasm were identified with DAPI and CellMask DeepRed fluorescence (S9 Fig). Afterwards the fluorescence volume of green-stained (total bacteria volume) and red-stained (external bacteria volume) were calculated and used as reference of bacterial biomass. Using these values, the relative amount of internalized and external bacteria was finally calculated. The statistical significance was determined by two-way ANOVA test (*p < 0.05).

### Antimicrobial assay

Bacteria grown in GC-1% v/v Isovitalex medium until early exponential phase, were suspended in the same liquid medium to a concentration of 10^5^ cfu/mL. To assess the antimicrobial activity of the peptide LL-37 (Sigma Aldrich) 180 μL of bacterial suspension were added to each well of a sterile 96-well plate containing 20 μL of peptide solution at several concentrations that ranged from 50 μM to 1.5 μM, in order to have a final concentration from 5 μM to 0.15 μM. In experiments with the Human Neutrophil Peptide-1 (HNP-1, Sigma Aldrich) and the Human β-Defensin-1 (HBD-1, Sigma Aldrich), 25 μL of bacterial suspension were added to the same volume of peptide, suspended in order to have final peptide concentrations ranging from 5 μM to 0.078 μM and from 2.4 μM to 0.075 μM, respectively. The samples were then incubated at 37°C for 1 h. In competition experiments, 20 μM polyP (Graham’s salt, Merck) or 20 μM DNA (deoxyribonucleic acid, low molecular weight from salmon sperm, Sigma Aldrich) were added to the incubation mixture. The activity of the peptides was represented as the minimum bactericidal concentration (MBC), defined as the lowest peptide concentration where bacterial growth was not detected. MBC values were determined by plating serial dilutions of the reaction mixture on GC-1% v/v Isovitalex agar plates and enumerating the cfu on day after. The percentage of surviving bacteria was expressed as cfu after peptide incubation divided by cfu after control incubation and multiplied by 100, according to Ong and colleagues [60].

### Quantitative real time PCR (qRT-PCR)

*N*. *gonorrhoeae* cultures were grown in 10 mL of GC-1% v/v Isovitalex liquid medium up to the exponential phase. Volume of the bacterial culture, corresponding to 1 OD, was then poured onto the same volume of frozen medium to immediately chill the culture and stop transcriptional changes. Cells were then harvested by centrifugation at 8000 x *g* for 10 min. Total RNA was isolated using a RNeasy Mini kit (Qiagen) as described by the manufacturer. A second step to remove residual DNA was performed by DNase treatment using RQ1 RNase-free DNase (Promega), for 1 h at 37°C and followed by a second RNA purification carried out with the RNeasy Mini kit. RNA was quantified using a Nanodrop 1000 spectrophotometer and its quality was assessed by gel electrophoresis, whereas the absence of residual DNA was confirmed by PCR, amplifying the *ppx* gene with the primers ppxKOextR/ppxWTF (Table 3). Two micrograms of total RNA were reverse-transcribed for cDNA synthesis using random hexamer primers and SuperScript II RT (Invitrogen), following manufacturer’s recommendations.

Quantitative RT-PCR was done in triplicate for each sample in a 25 μL reaction volumes using Platinum SYBR Green qPCR SuperMix-UDG with Rox (Thermo Fisher) according to the manufacturer’s instructions and containing 1 ng of cDNA and 2.5 μM of gene-specific primers (Table 3). Amplification and detection of specific products were performed with a Mx3000P Real-Time PCR system (Stratagene) using the following cycling parameters: 95°C for 10 min, followed by 40 cycles of 95°C for 30 s, 55°C for 30 s and 72°C for 30 s then ending with a dissociation curve analysis. The *16S RNA* gene was used as the endogenous reference control and the relative transcript change was determined using the 2^-ΔΔCt^ relative quantification method [61].

### Bioinformatic analysis

Nucleotide sequences of the *ppk1*, *ppk2* and *ppx* were extracted from the genomes of the four gonococcal strains under investigation. The alignment of the amino acid sequences was performed using the CLUSTAL Omega algorithm incorporated within the Geneious software (Biomatters) [62].

## Supporting information

S1 Fig Binding of DNA to the surface of *Neisseria gonorrhoeae* F62. The immunofluorescence images show bacterial nucleoid in blue stained with DAPI and DNA binding to bacterial surface in purple. Panel **A** represents the negative control stained with the fluorescent secondary antibody only. Panel **B** describes DNA binding to *N*. *gonorrhoeae* F62 surface.
(TIF)

S2 Fig LOS sialylation does not compete with polyP for the protection from cathelicidin LL-37. The survival of *N*. *gonorrhoeae* F62, grown without (**A**) or in the presence (**B**) of 50 μg/mL of CMP-NANA, was evaluated by incubating 10^5^ cfu/mL with increasing concentration of the peptides for 2 h at 37°C. The ability of the polyP to restore survival was evaluated by including in the bacterial suspension 20 μM polyP (broken line). The number of surviving bacteria was counted by plating serial dilution of incubated bacterial suspensions and compared to the number of bacteria in the control. The vertical bars represent the standard deviation of the mean of two independent experiments.
(TIF)

S3 Fig Alpha- and beta-defensins are not relevant for polyP dependent gonococcal infection. The survival of *N*. *gonorrhoeae* with Human Neutrophils Peptide-1 (HNP-1) (**A**) and Human β-defensin-1 (HBD-1) (**B**) was evaluated by incubating 10^5^ cfu/mL with increasing concentration of the peptides for 1 h at 37°C. The number of surviving bacteria was counted by plating serial dilution of incubated bacterial suspensions and relating it to the number of bacteria in the control. The error bars represent the standard deviation of the mean of two independent experiments.
(TIF)

S4 Fig The polyP pseudo-capsule is not detected on the surface of PorB.1A strains. Flow cytometric analyses were carried out on SK92-679, WHO-N and WHO-F to detect polyP pseudo-capsule. Shaded grey profiles represent the negative controls stained with only the fluorescent secondary antibody instead, the purple pics exhibit the reaction with the primary antibody against the His-tag of the recombinant *Ec*PPXc.
(TIF)

S5 Fig PorB.1A *N*. *gonorrhoeae* SK92-679 serum resistance is independent from polyP pseudo-capsule. *N*. *gonorrhoeae* SK92-679 was incubated with 25% NHS (filled bar) and with NHS complemented with 50 μM polyP (stripped bar). Results of serum resistance assays are specified as percentage of survival calculated as the ratio of cfu after incubation with NHS over cfu incubated with heat inactivated NHS. Vertical bars show the standard deviation.
(TIF)

S6 Fig Multiple sequence alignment of polyP enzymes carried by the four *N*. *gonorrhoeae* strains confirms the high level of sequence conservation. In the three panels A, B and C, the alignment of amino acid sequence of the polyphosphate kinase 1 (PPK1), polyphosphate kinase 2 (PPK2) and exopolyphosphatase (PPX) is respectively depicted. The consensus sequence is shown above the aligned sequences and the colored bar represents the identity across all sequences: in green 100% identity and in yellow lower identity. Within the multiple sequence alignment, conserved amino acids are represented by dots and mismatched amino acids are indicated.
(TIF)

S7 Fig PolyP pseudo-capsule production does not correlate with transcription level of polyP metabolic enzymes. *ppk1*, *ppk2 and ppx* RNA levels were quantified by qRT-PCR and relative expression levels were determined normalizing to *16S-rRNA*. Results are represented relatively to FA1090 enzymes transcription level.
(TIF)

S8 Fig Purity and stability of the recombinant C-terminal domain of PPX (*Ec*PPXc). **A**. The *Ec*PPXc is resolved through the lanes 2–7 of a 4–12% polyacrylamide gel. The unique band of apparent molecular weight 24 kDa, indicates the purity, integrity and stability of the protein after five freeze-thaw cycles. **B**. The analytical size exclusion chromatography (SE-UPLC) profile shows a retention time between picA (158 kDa) and B (44 kDa), confirming its expected dimeric structure [23] and a high purity level.
(TIF)

S9 Fig Image captured by Opera Phenix. Internalized gonococci are stained in green while externally-associated bacteria are visualized in yellow. dHL60 cells are visualized with CellMask DeepRed.
(TIF)
