# Supplementary figures and images for: Functional characterization of the gonococcal polyphosphate pseudo-capsule
Source: PLoS Pathog. 2023 May 22;19(5):e1011400. doi: 10.1371/journal.ppat.1011400 (PMC10237660; doi:10.1371/journal.ppat.1011400)

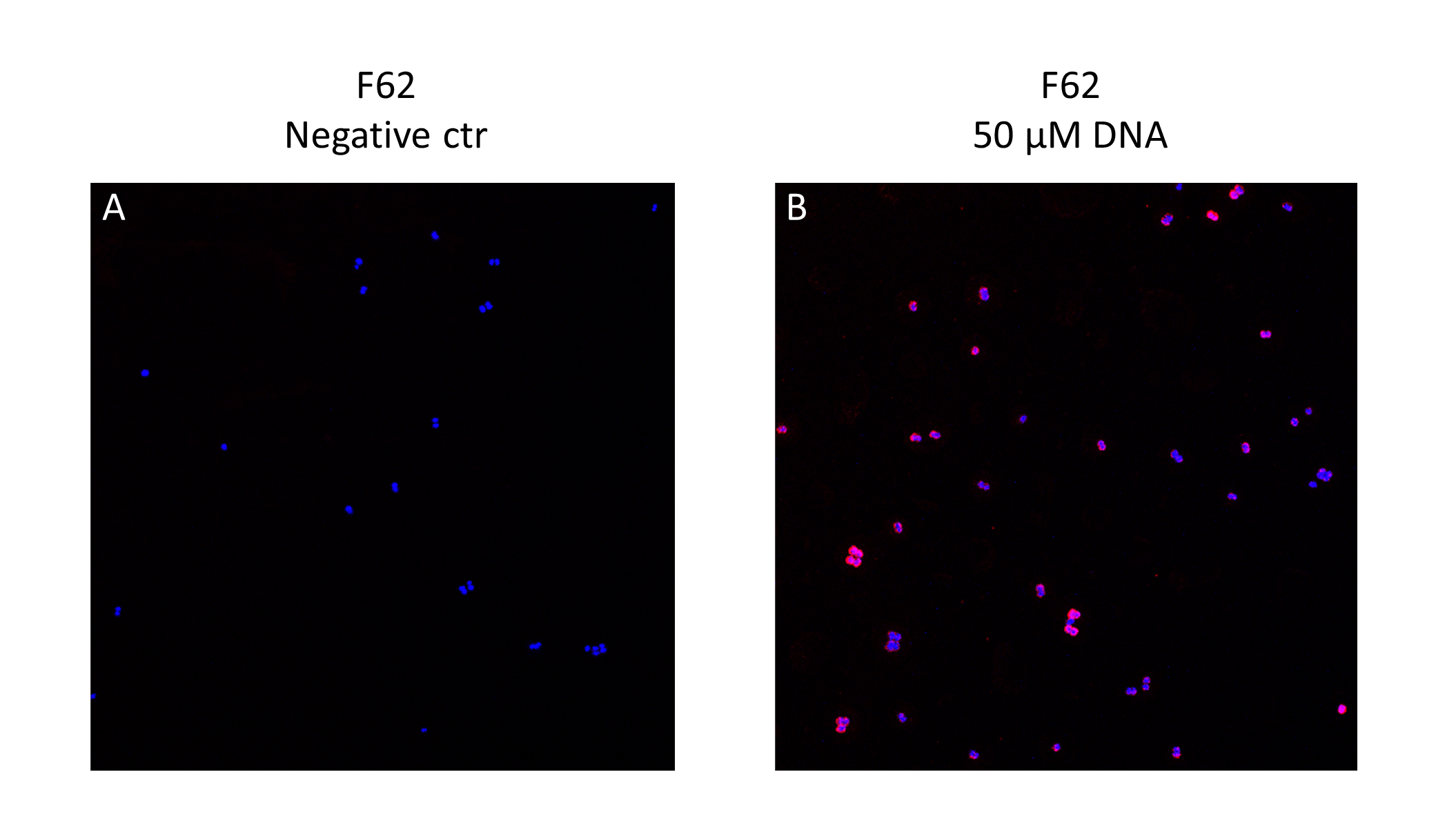

Supplement: S1 Fig — The immunofluorescence images show bacterial nucleoid in blue stained with DAPI and DNA binding to bacterial surface in purple. Panel A represents the negative control stained with the fluorescent secondary antibody only. Panel B describes DNA binding to N. gonorrhoeae F62 surface. (TIF) [file ppat.1011400.s001.tif]

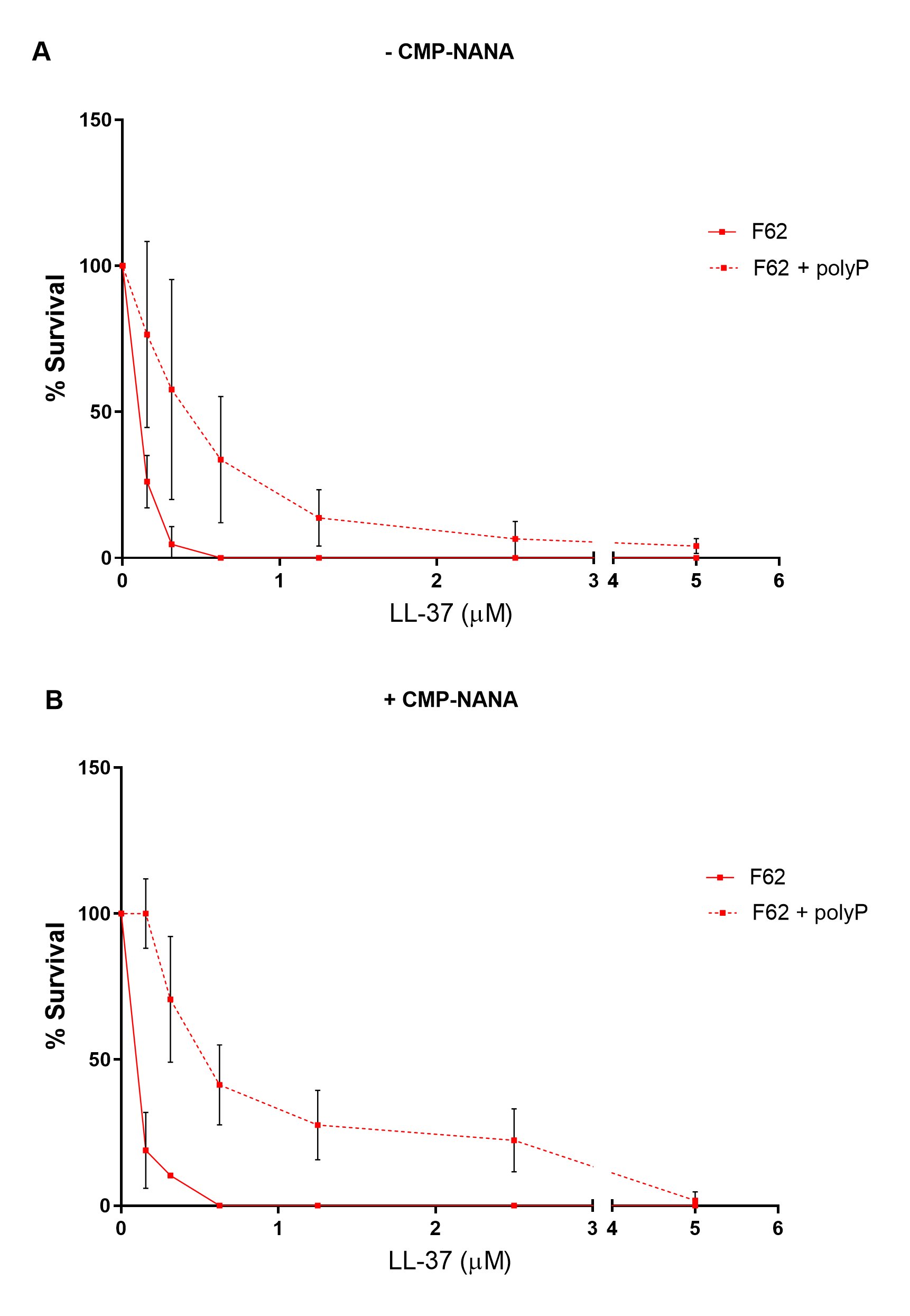

Supplement: S2 Fig — The survival of N. gonorrhoeae F62, grown without (A) or in the presence (B) of 50 μg/mL of CMP-NANA, was evaluated by incubating 105 cfu/mL with increasing concentration of the peptides for 2 h at 37°C. The ability of the polyP to restore survival was evaluated by including in the bacterial suspension 20 μM polyP (broken line). The number of surviving bacteria was counted by plating serial dilution of incubated bacterial suspensions and compared to the number of bacteria in the control. The vertical bars represent the standard deviation of the mean of two independent experiments. (TIF) [file ppat.1011400.s002.tif]

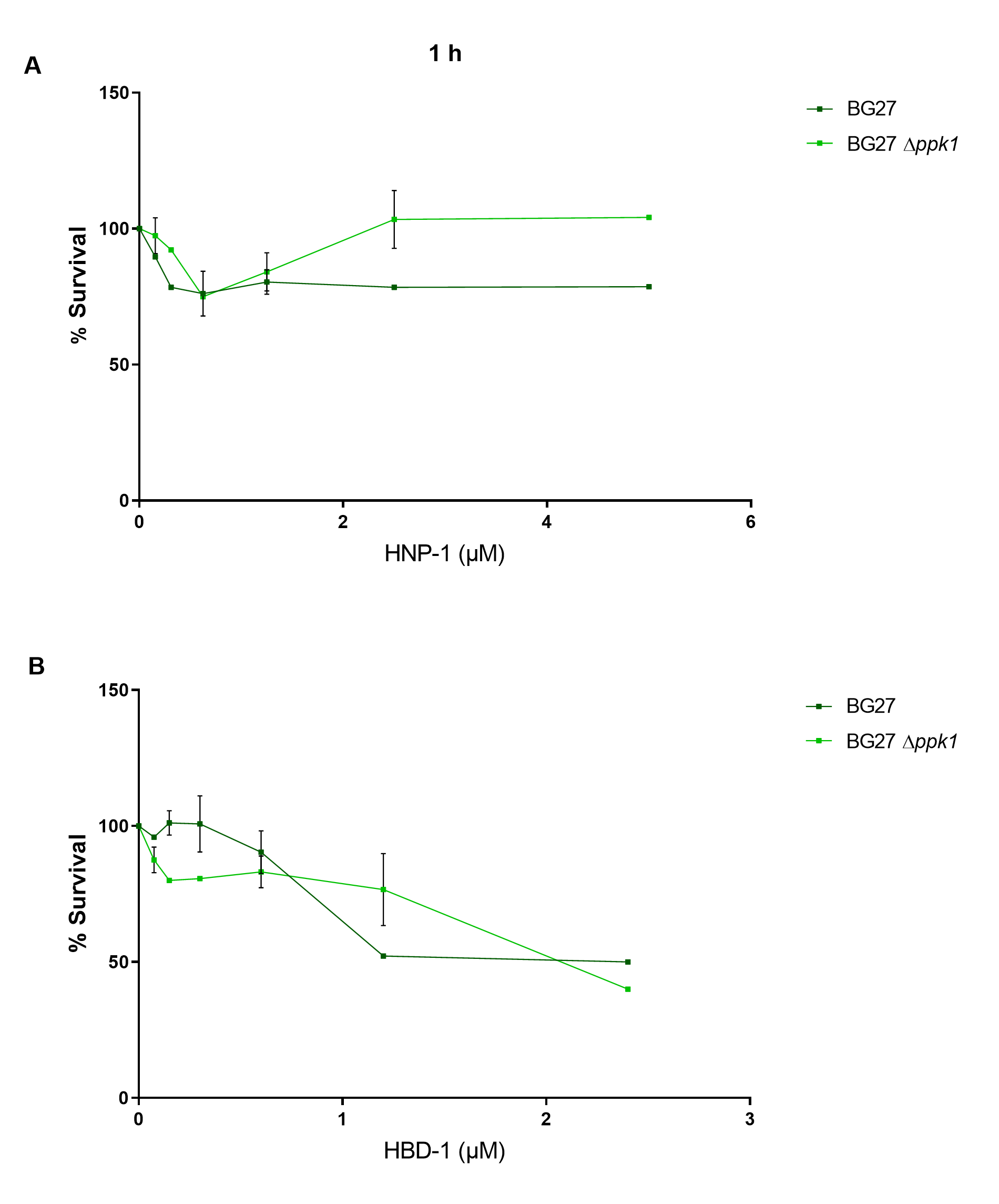

Supplement: S3 Fig — The survival of N. gonorrhoeae with Human Neutrophils Peptide-1 (HNP-1) (A) and Human β-defensin-1 (HBD-1) (B) was evaluated by incubating 105 cfu/mL with increasing concentration of the peptides for 1 h at 37°C. The number of surviving bacteria was counted by plating serial dilution of incubated bacterial suspensions and relating it to the number of bacteria in the control. The error bars represent the standard deviation of the mean of two independent experiments. (TIF) [file ppat.1011400.s003.tif]

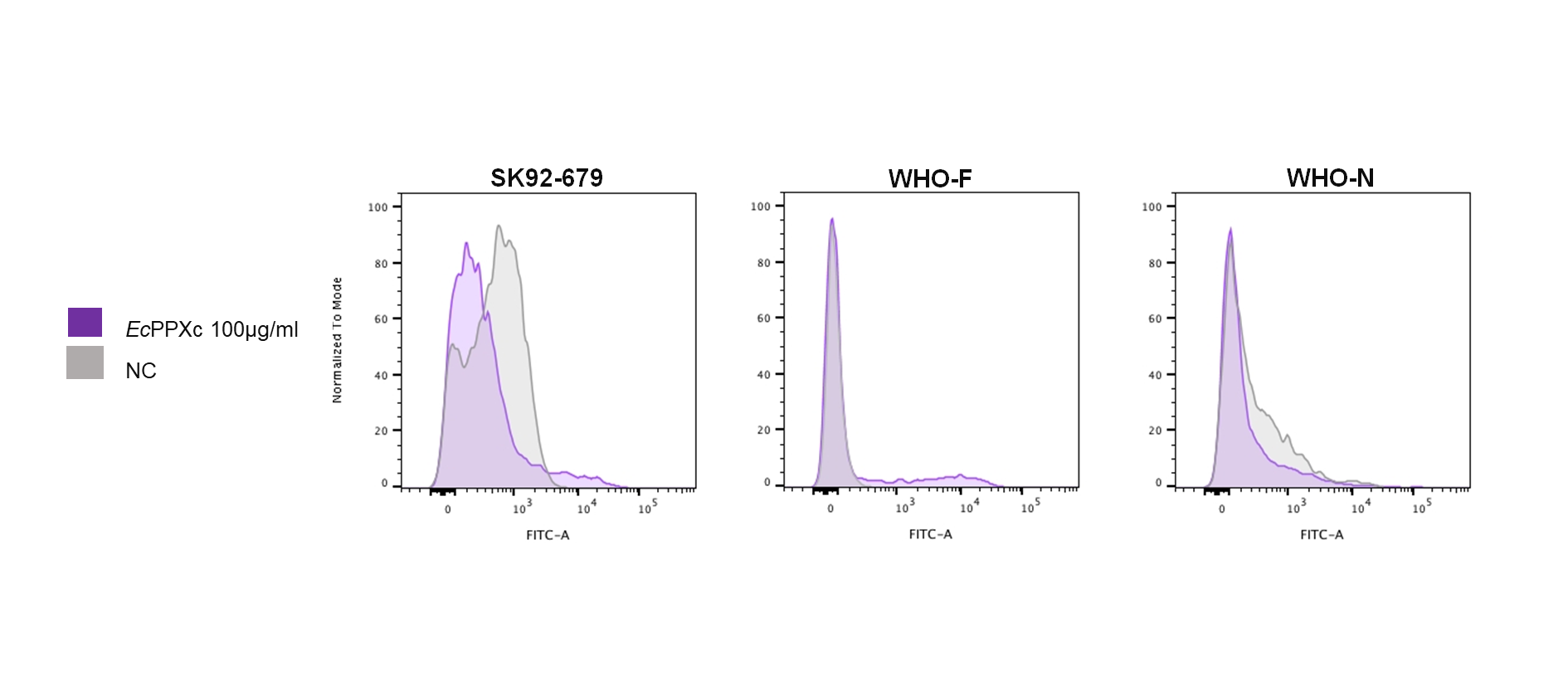

Supplement: S4 Fig — Flow cytometric analyses were carried out on SK92-679, WHO-N and WHO-F to detect polyP pseudo-capsule. Shaded grey profiles represent the negative controls stained with only the fluorescent secondary antibody instead, the purple pics exhibit the reaction with the primary antibody against the His-tag of the recombinant EcPPXc. (TIF) [file ppat.1011400.s004.tif]

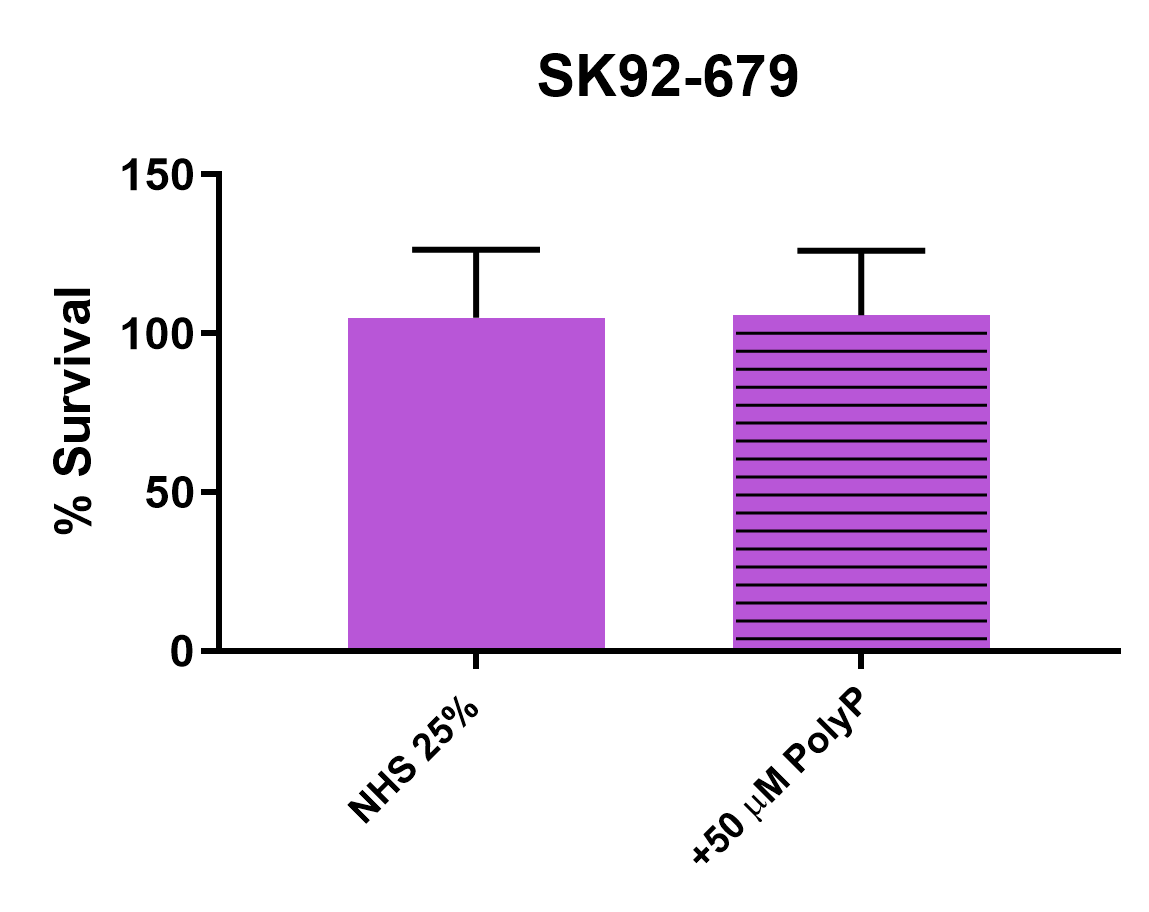

Supplement: S5 Fig — N. gonorrhoeae SK92-679 was incubated with 25% NHS (filled bar) and with NHS complemented with 50 μM polyP (stripped bar). Results of serum resistance assays are specified as percentage of survival calculated as the ratio of cfu after incubation with NHS over cfu incubated with heat inactivated NHS. Vertical bars show the standard deviation. (TIF) [file ppat.1011400.s005.tif]

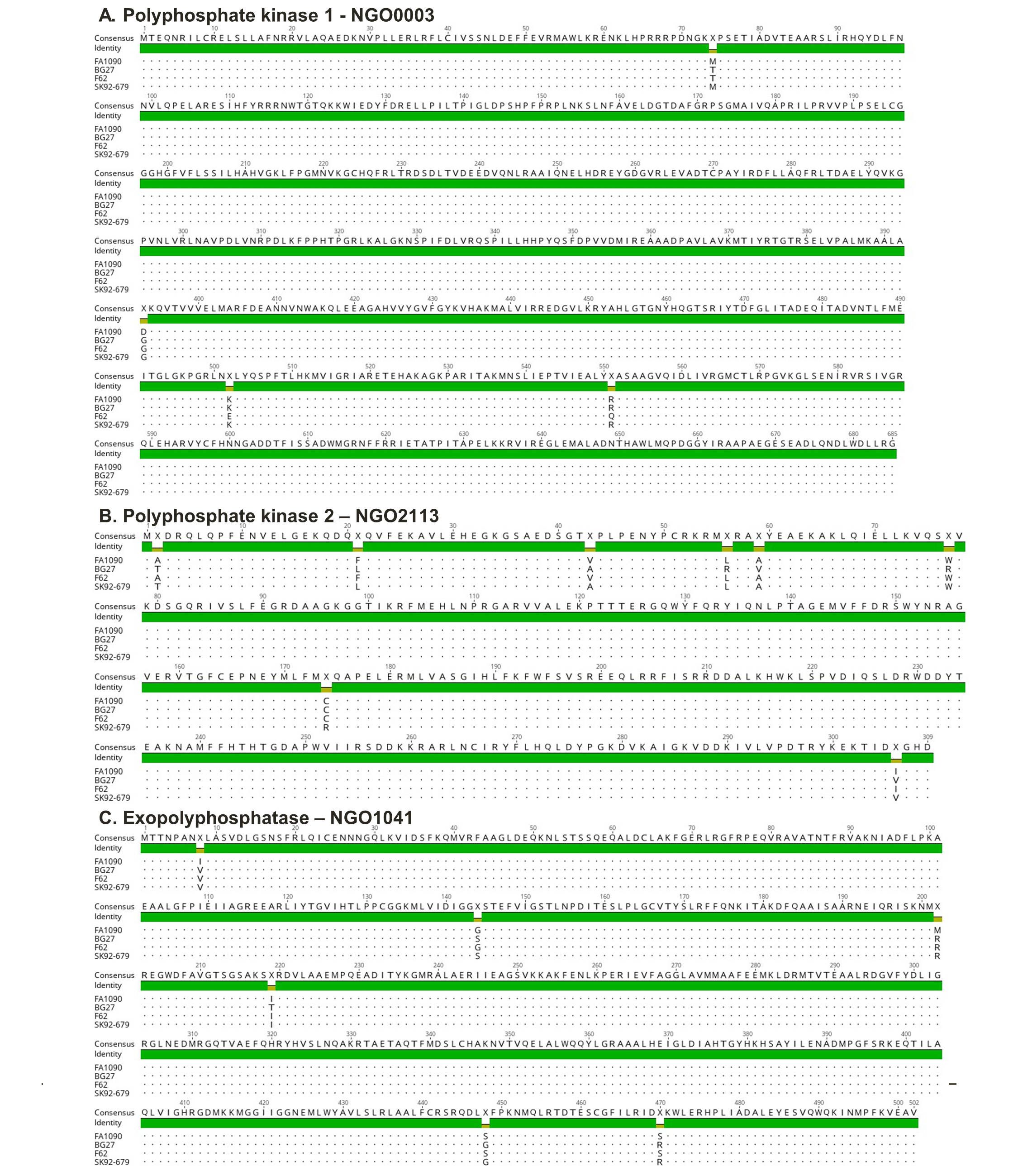

Supplement: S6 Fig — In the three panels A, B and C, the alignment of amino acid sequence of the polyphosphate kinase 1 (PPK1), polyphosphate kinase 2 (PPK2) and exopolyphosphatase (PPX) is respectively depicted. The consensus sequence is shown above the aligned sequences and the colored bar represents the identity across all sequences: in green 100% identity and in yellow lower identity. Within the multiple sequence alignment, conserved amino acids are represented by dots and mismatched amino acids are indicated. (TIF) [file ppat.1011400.s006.tif]

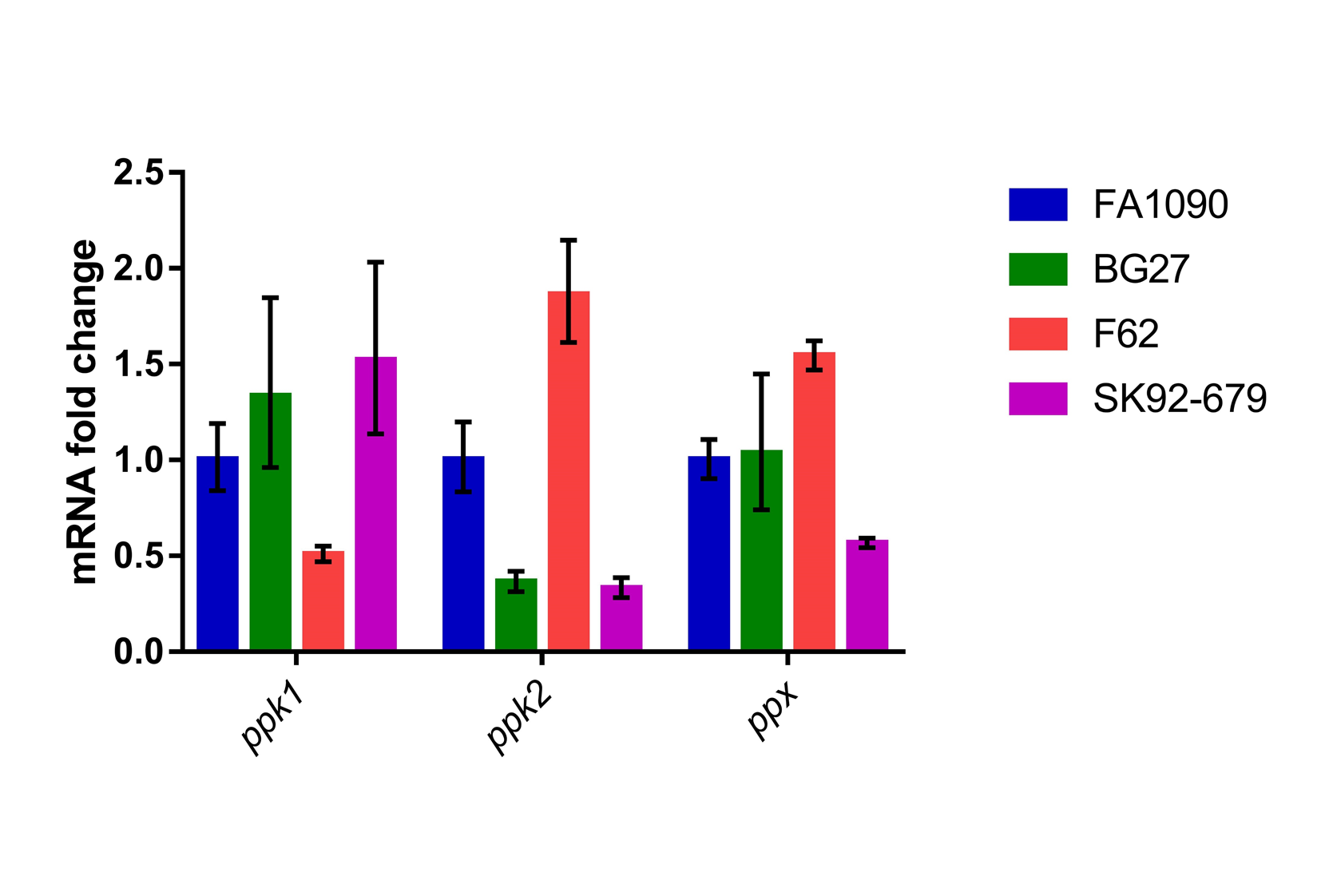

Supplement: S7 Fig — ppk1, ppk2 and ppx RNA levels were quantified by qRT-PCR and relative expression levels were determined normalizing to 16S-rRNA. Results are represented relatively to FA1090 enzymes transcription level. (TIF) [file ppat.1011400.s007.tif]

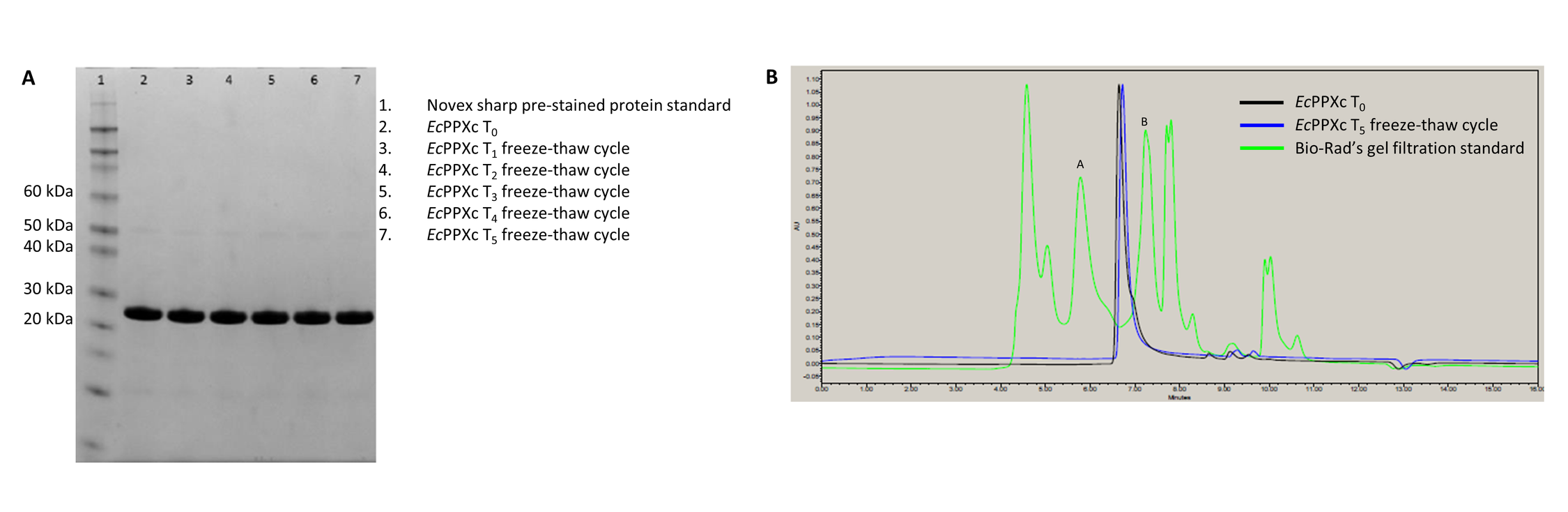

Supplement: S8 Fig — A. The EcPPXc is resolved through the lanes 2–7 of a 4–12% polyacrylamide gel. The unique band of apparent molecular weight 24 kDa, indicates the purity, integrity and stability of the protein after five freeze-thaw cycles. B. The analytical size exclusion chromatography (SE-UPLC) profile shows a retention time between picA (158 kDa) and B (44 kDa), confirming its expected dimeric structure [23] and a high purity level. (TIF) [file ppat.1011400.s008.tif]

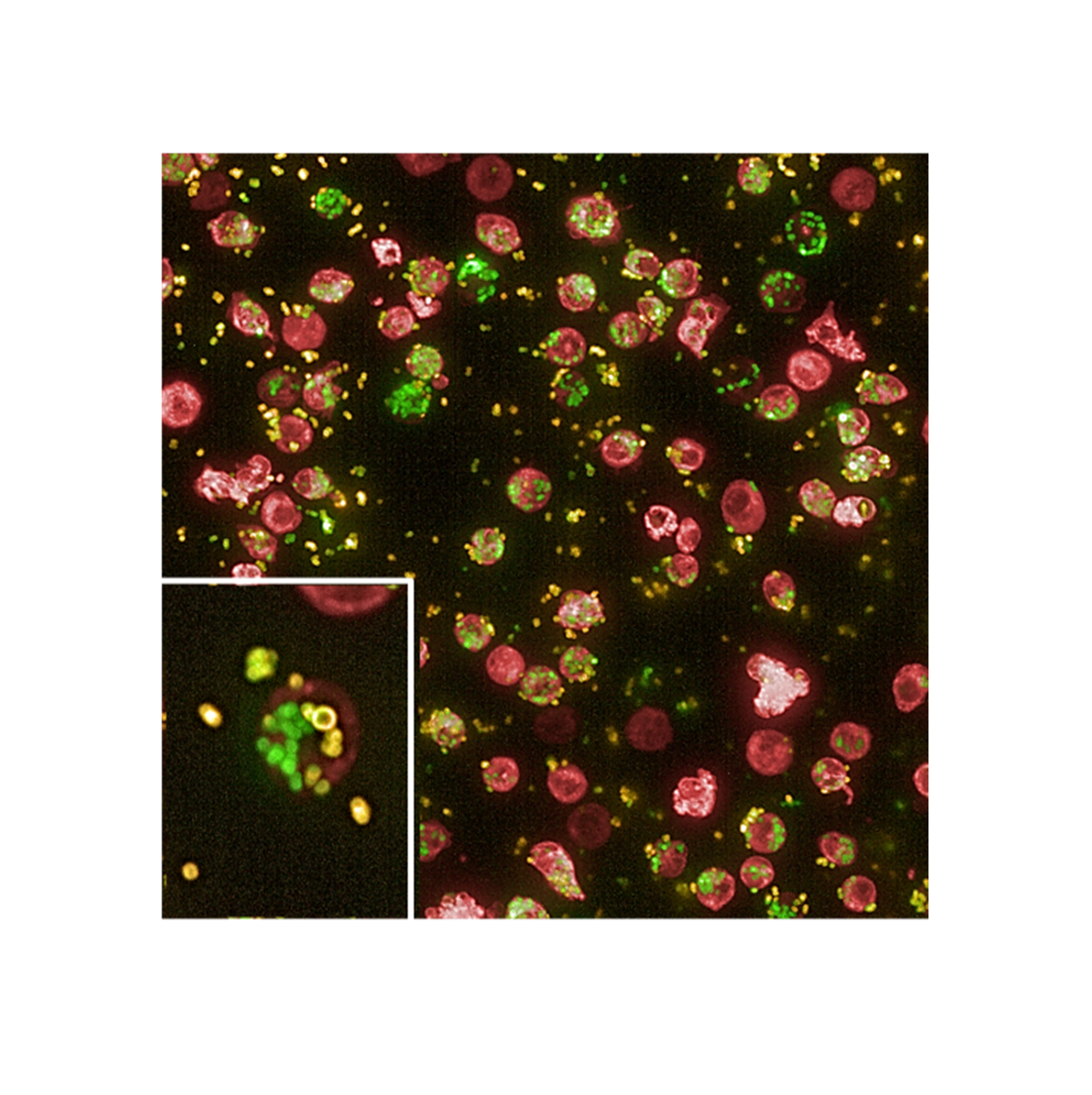

Supplement: S9 Fig — Internalized gonococci are stained in green while externally-associated bacteria are visualized in yellow. dHL60 cells are visualized with CellMask DeepRed. (TIF) [file ppat.1011400.s009.tif]
